# A comprehensive review of the physiology and evidence base to guide the use of ergogenic and medical supplements for enhanced cycling performance

**DOI:** 10.1080/15502783.2026.2630487

**Published:** 2026-02-13

**Authors:** Andrew Rowland, Sophie Edwards, Gorka Prieto-Bellver, Bradley Menz, Angela Rowland, Erik Cornelisse, Christos S Karapetis, Matthew P Wallen, Ashley M Hopkins

**Affiliations:** a College of Medicine and Public Health, Flinders University, Adelaide, South Australia, Australia; b Performance and Sport Rehabilitation Laboratory, Faculty of Sport Sciences, University of Castilla-La Mancha, Toledo, Spain; c Clinical Toxicology Service, Royal Adelaide Hospital, Adelaide, South Australia, Australia; d College of Nursing and Health Science, Flinders University, Adelaide, South Australia, Australia

**Keywords:** Cycling, ergogenic supplements, medical supplements, metabolic testing, skeletal energy metabolism

## Abstract

**Background:**

A growing body of evidence supports the use of supplements to enhance cycling performance through both direct and indirect mechanisms.

**Methods:**

This review was informed by a structured literature search conducted in PubMed, Scopus and Web of Science for peer-reviewed studies published up to May 2025. Studies were included if they involved human participants, were published in English and evaluated outcomes related to endurance performance, recovery or physiological function.

**Results:**

Direct enhancement with ergogenic supplements is primarily achieved via modulation of skeletal muscle energy metabolism. During exercise, adenosine triphosphate (ATP) resynthesis is driven by the phosphagen system, glycolysis, oxidative phosphorylation and beta-oxidation, with each system contributing according to the intensity and duration of the effort. Supplements such as beta-alanine, caffeine, carbohydrates, carnitine, creatine monohydrate, dietary nitrates, electrolytes, exogenous ketones, N-acetylcysteine and sodium bicarbonate support these energy systems by improving substrate utilization, buffering capacity, energy availability or resistance to fatigue. In addition to ergogenic supplements that directly enhance performance, medical supplements play an important indirect role by supporting bone health, connective tissue integrity, inflammation management, micronutrient status, muscle repair and gut function. Evidence-based options for cyclists include calcium, cherry juice, collagen, curcumin, iron, multivitamins, omega-3 fatty acids, pickle juice, probiotics, protein, vitamin C, vitamin D and zinc. Each contribute to either improved recovery, immune support or long-term physiological adaptation. Evidence quality varied substantially across supplements, with strongest support for Australian Institute of Sport (AIS) Group A compounds. The integration of physiological testing including assessments of maximal oxygen consumption (VO_2_max), lactate threshold, metabolic substrate utilization and blood biomarkers may inform the development of individualized supplementation strategies tailored to training demands and competitive goals.

**Conclusions:**

This evidence-informed approach underscores the synergistic relationship between nutrition, training and performance optimization in cycling. Future research should explore personalized nutrition frameworks, interactions between multi-supplement protocols and the molecular mechanisms underpinning adaptation to endurance training and nutritional interventions.

## 1. Introduction

A growing body of evidence supports the use of supplements to enhance cycling performance. These supplements are broadly classified as ergogenic (direct) supplements, which acutely enhance performance, and medical (indirect) supplements that enable consistent training and improved physical resilience. Ergogenic supplements can increase power output, endurance and fatigue resistance across both high intensity and endurance cycling disciplines [1]. Although ergogenic supplements vary in their mechanisms of action, they commonly target skeletal muscle energy metabolism and buffering capacity. Sustained muscular contraction requires a continuous supply of adenosine triphosphate (ATP), which is regenerated through four primary metabolic pathways: the phosphagen system, glycolysis, oxidative phosphorylation and beta-oxidation [2]. Medical supplements similarly function via various mechanisms to support overall health, recovery, immune function and long-term physiological adaptation. Medical supplements typically address micronutrient deficiencies, enhance musculoskeletal integrity, reduce inflammation or promote effective repair processes following training. By maintaining physiological systems that underpin consistent and high-quality training, medical supplements may play an important supporting role in sustained performance outcomes.

To guide the safe and effective use of ergogenic and medical supplements, classification systems such as the Australian Institute of Sport (AIS) ABCD framework have been developed. These systems provide evidence-based recommendations for athletes and practitioners. AIS Group A supplements are supported by strong evidence and approved for use in evidence-based protocols. AIS Group B supplements have emerging but incomplete evidence and are typically recommended for use only under supervision in research or case-managed scenarios. AIS Group C supplements lack sufficient scientific evidence to support benefits for performance or recovery. Their use is not recommended in routine practice and should be restricted to research settings. AIS Group D supplements are banned or pose a high risk of contamination with prohibited substances and are therefore considered unsafe and inappropriate for competitive athletes. Working within the broad guidance provided by the AIS framework, there is increasing recognition of the need to personalise supplement strategies based on individual factors such as sex, age, hormonal status, genetic predispositions and gut microbiota composition, which may influence both efficacy and safety. However, evidence supporting individualised supplementation strategies remains limited and largely extrapolated.

While not the focus of this review, it is worth noting that many AIS Group A and B supplements are used in competition, so compliance with anti-doping rules is important. The Union Cycliste Internationale (UCI) enforces World Anti-Doping Agency (WADA) rules through the International Testing Agency (ITA), which manages testing and results. Under WADA strict liability, athletes are fully responsible for any banned substances in their system, regardless of intent. Contaminated or mislabelled supplements pose a doping risk, so athletes should use products tested by reputable third-party programs. WADA recognises certifications from ISO/IEC 17025-accredited labs, which screen for banned substances. Similarly, many AIS Group A and B supplements can impact health beyond performance, including the risk of adverse effects or interactions with medications. Some ingredients may alter exposure to or effectiveness of prescription medicines, potentially reducing efficacy or increasing toxicity. These risks are well-documented, particularly for individuals managing chronic conditions or using multiple medications. To minimise harm, athletes taking any prescription medicines should consult a qualified health professional before using supplements, prioritise products with transparent labelling and third-party testing, and closely monitor for side effects or unexpected reactions.

Understanding the physiological basis that underpins the performance enhancement achieved with these supplements provides a foundation for their strategic and individualised application in training and competition. The objective of this review is to provide an overview of the key steps within metabolic pathways underlying skeletal muscle energy production as a basis to understand the mechanism of ergogenic supplements. The physiological and evidence-based rationale for ergogenic and medical supplements in cycling is then considered in the context of enhancing performance across different disciplines. We also explore how physiological testing including VO₂max, lactate profiling and metabolic assessments can help inform individualised supplementation strategies tailored to specific performance goals.

## 2. Methods

A structured narrative approach was employed to review the physiology and evidence base for the use of ergogenic and medical supplements relevant to cycling performance. Literature searches regarding evidence for supplements were conducted in PubMed (January 2000 to May 202was 5), Scopus (January 2000 to May 2025), and Web of Science (January 2000 to May 2025). To capture foundational knowledge in muscle bioenergetics and exercise physiology, additional literature dating from January 1960 to May 2025 was considered. This extended date range was chosen to include seminal work on glycolysis, oxidative phosphorylation, acid–base regulation, substrate metabolism and fatigue mechanisms that underpin contemporary supplementation strategies.Search terms included combinations of keywords and Boolean operators related to cycling, supplementation and performance. An example search string used in PubMed was: (“cycling” OR “cyclist” OR “endurance exercise”) AND (“supplement” OR “ergogenic aid*” OR “nutritional supplement*”) AND (“performance” OR “power output” OR “time trial” OR “fatigue” OR “recovery”)*, with additional searches incorporating specific supplement names (e.g. “beta-alanine,” “caffeine,” “dietary nitrate,” “creatine,” “omega-3,” “curcumin,” “carnitine”). Equivalent search strategies were adapted for Scopus and Web of Science.

Peer-reviewed original research articles, meta-analyses and systematic reviews written in English were included. Studies were excluded if they were animal studies, lacked exercise-relevant endpoints or were non-peer-reviewed sources. A subset of key recent systematic reviews and meta-analyses were used to conduct a structured synthesis evaluating the strength and consistency of evidence for AIS Group A and B supplements with respect to cycling performance. The strength of evidence for supplements in cycling was evaluated using the NHMRC Body of Evidence framework, informed by GRADE principles. Systematic reviews and meta-analyses of RCTs were prioritised, with narrative reviews considered only when higher-level evidence was lacking. Evidence was first classified by study design (systematic reviews of RCTs starting as high), then downgraded or upgraded based on risk of bias (e.g. blinding, allocation concealment, sample size), consistency across studies, directness to the research question, precision of effect estimates and potential publication bias. Certainty was expressed as High, Moderate, Low or Very Low, while the NHMRC framework provided complementary ratings (Grades A–D) based on evidence base, consistency, impact, generalisability and applicability.

## 3. Skeletal energy production: the basis for ergogenic supplements

Skeletal muscle energy production is sustained by a network of interrelated metabolic pathways that regenerate ATP. These pathways are activated to varying degrees based on the intensity and duration of exercise. Understanding their regulation and contribution during different cycling demands provides a foundation for the use of ergogenic supplements to modulate substrate availability, buffering capacity and mitochondrial efficiency.

### 3.1. The phosphagen system: rapid ATP regeneration

The phosphagen system dominates energy provision during the first 5 to 15 seconds of maximal effort [3]. This anaerobic mechanism relies on phosphocreatine (PCr) stored in muscle fibres. The key reaction is catalysed by creatine kinase (CK), which transfers a phosphate group from PCr to adenosine diphosphate (ADP), forming ATP:

$$\mathrm{Phosphocreatine}+\mathrm{ADP}\overset{\mathrm{CK}}{\to }\mathrm{Creatine}+\mathrm{ATP}$$

While this system supplies ATP almost instantaneously, its capacity is limited by the size of intramuscular PCr stores. The abundance of PCr stored in skeletal muscle is therefore a primary determinant of maximal power output and the duration over which a maximal effort can be sustained. This physiological limitation forms the mechanistic rationale for the use of supplements, such as creatine, which increase intramuscular PCr abundance and may thereby enhance peak power and extend the duration of maximal effort. Typically, 1 mole of PCr yields 1 mole of ATP, with no net production of lactate or requirement for oxygen. It serves primarily as a temporal buffer until slower-acting energy pathways are initiated.

### 3.2. Glycolysis: anaerobic ATP production

Glycolysis involves the anaerobic breakdown of glucose or glycogen to form pyruvate or lactate, generating ATP and NADH in the cytoplasm. It becomes a dominant pathway during moderate to high-intensity efforts lasting up to two minutes. When glucose, sourced from the bloodstream, is utilised the pathway yields a net production of 2 ATP per glucose molecule. However, when glycogen stored in the muscle is used, one ATP is spared in the phosphorylation step, yielding 3 ATP per glycogen monomer. This ten-step enzymatic cascade is mediated by several key enzymes, including hexokinase, phosphofructokinase-1 (PFK-1) and pyruvate kinase. Of these enzymes, PFK-1 is the most critical regulatory of glycolysis. It catalyses the rate-limiting, committed step of glycolysis, making it a critical control point for cellular energy production [4]:

$$\mathrm{Fructose}\;6\;\mathrm{phosphate}+\mathrm{ATP}\overset{\mathrm{PFK}-1}{\to }\mathrm{Fructose}\;1,6\;\mathrm{bisphosphate}+\mathrm{ADP}$$

This irreversible phosphorylation commits the glucose molecule to proceed through glycolysis. As the rate-limiting enzyme in glycolysis, PFK-1 regulates glycolytic flux based on energy demand, coordinates with other metabolic pathways (e.g. gluconeogenesis), enables rapid energy production during high-intensity exercise and is a target for regulation by hormonal signals such as insulin and glucagon [4]. PFK-1 activity is highly sensitive to intracellular pH. Under acidic conditions, such as intense anaerobic exercise, PFK-1 activity is markedly inhibited (to ~50% at pH 7.2), thereby slowing glycolytic flux. Accordingly, intracellular pH serves as a critical modulator of PFK-1 and cellular energy metabolism [5]. This pH-dependent sensitivity of key enzymes involved in glycolysis, such as PFK-1, underpins the mechanistic rationale for the use of supplements including beta-alanine and sodium bicarbonate that buffer intracellular and extracellular pH, thereby preserving glycolytic flux and delaying the onset of fatigue during high-intensity efforts [6].

In the absence of oxygen, lactate dehydrogenase A (LDH-A) reduces pyruvate to lactate, regenerating NAD⁺ to sustain flux through glycolysis. The ratio of lactate to pyruvate formed during glycolysis, termed the lactate-to-pyruvate (L/P) ratio, reflects the cytosolic redox state and the balance between anaerobic and aerobic metabolism [7]. Under resting or low-intensity exercise conditions, the L/P ratio is typically around 10:1, indicating efficient mitochondrial oxidation of NADH and predominant aerobic metabolism. During high-intensity exercise, reduced oxygen availability limits oxidative phosphorylation, causing the ratio to rise to 50:1 or higher. This shift results from increased conversion of pyruvate to lactate via LDH-A to regenerate NAD⁺, thereby sustaining glycolytic ATP production [8]. The elevated L/P ratio thus serves as a metabolic adaptation to support energy demands under anaerobic conditions. Importantly, contemporary lactate shuttle theory recognises lactate not as a metabolic dead-end or primary cause of fatigue, but as a key intermediary metabolite that can be transported between fibres and tissues and oxidised by mitochondria as an energy substrate, particularly in well-trained muscle [9]. Thus, increased lactate production during high-intensity cycling reflects a regulated redistribution of carbon and reducing equivalents rather than a failure of oxidative metabolism. Despite its inefficiency relative to oxidative pathways, glycolysis provides a rapid ATP supply and is vital in cycling disciplines requiring repeated sprinting or bursts of effort.

### 3.3. Oxidative phosphorylation: the endurance engine

Oxidative phosphorylation is the most efficient ATP-generating process and is fundamental to endurance cycling performance [10]. It involves the mitochondrial oxidation of carbohydrates, lipids and proteins, ultimately reducing molecular oxygen to water through the electron transport chain (ETC). The process begins with the conversion of pyruvate, typically the end-product of glycolysis, into acetyl-CoA via the pyruvate dehydrogenase complex (PDC). Acetyl-CoA then enters the tricarboxylic acid (TCA) cycle, where sequential enzymatic reactions generate NADH and FADH₂, which subsequently donate electrons to the ETC. Key regulatory enzymes, such as isocitrate dehydrogenase and *α*-ketoglutarate dehydrogenase, modulate carbon flux through the TCA cycle and are sensitive to cellular energy status and substrate levels [10]. As electrons traverse the ETC, a proton gradient is established across the inner mitochondrial membrane, driving ATP synthesis via ATP synthase. Complete oxidation of one glucose molecule through this integrated system yields approximately 30 to 32 ATP molecules. Actual ATP yield varies with mitochondrial efficiency, proton leak and NADH shuttle mechanisms [11]. Although slower than glycolysis or PCr hydrolysis, oxidative phosphorylation is capable of sustaining ATP production for prolonged durations, constrained chiefly by oxygen availability and the supply of metabolic substrates [12].

Given the importance of oxidative phosphorylation to cycling performance, several supplements act by targeting specific steps to enhance oxidative capacity. Dietary nitrates, for example, increase circulating nitric oxide concentrations, leading to vasodilation and enhanced nutrient delivery to muscles, thereby improving mitochondrial efficiency and oxygen utilisation [13]. Carbohydrates serve as a primary fuel for oxidative phosphorylation, while L-carnitine facilitates the transport of long-chain fatty acids into the mitochondria for *β*-oxidation, potentially increasing the availability of acetyl-CoA for the TCA cycle. Collectively, these strategies reflect a mechanistic rationale for using targeted supplementation to optimise oxidative metabolism, promote endurance performance, and safeguard physiological resilience in demanding training and environmental conditions.

### 3.4. Beta-oxidation: lipid-fuelled energy

During prolonged, low-intensity efforts, fatty acids become the predominant fuel source through the mitochondrial process known as beta-oxidation [14]. Here, long-chain fatty acids are transported into the mitochondrial matrix via carnitine acyltransferase I and II, then sequentially cleaved into two-carbon units (acetyl-CoA) by the beta-oxidation spiral [15]. Each cycle also generates 1 NADH and 1 FADH₂, feeding into the ETC. For instance, the oxidation of palmitate (C16:0) produces 8 acetyl-CoA, 7 NADH, and 7 FADH₂, which together yield approximately 106 ATP molecules, subtracting 2 ATP for activation. Despite this high yield, beta-oxidation is relatively slow and ultimately oxygen-dependent, making it unsuitable for rapid or high-intensity efforts. Nonetheless, its energy density makes it essential for ultra-endurance performance and during periods of glycogen depletion [14]. The reliance on fatty acid oxidation during prolonged, low-intensity exercise provides a mechanistic basis for ergogenic strategies that enhance mitochondrial lipid metabolism. Supplements such as L-carnitine have been proposed to augment fatty acid transport into the mitochondrial matrix via the carnitine shuttle, which may improve the efficiency of beta-oxidation.

### 3.5. Integration and ATP yields

The interplay between energy metabolism systems allows the body to adapt to varying intensities and cycling durations. The phosphagen system offers immediate energy but is quickly exhausted. Glycolysis provides a rapid but limited supply of ATP and contributes to acidosis under anaerobic conditions. Oxidative phosphorylation, although slower to engage, sustains long-term ATP production using both carbohydrate and fat substrates. Beta-oxidation is indispensable during prolonged activity but is restricted by mitochondrial oxygen supply and lipid mobilisation rates.

## 4. Ergogenic supplements to directly enhance cycling performance

The most widely used supplements for directly enhancing cycling performance are those permitted under WADA regulations and supported by robust scientific evidence regarding their safety and efficacy. These compounds are classified within Group A or Group B of the AIS ABCD system (Table 1) [1]. Their specific utility depends on the physiological demands of different cycling disciplines. When applied appropriately, considering individual training load, event characteristics and athlete physiology, these supplements can provide meaningful enhancements in both performance and recovery (Table 2). Of note, permitted ergogenic supplements are intended for oral administration only, consistent with physiological intake pathways and aligned with evidence-based protocols. Intravenous (IV) administration is restricted under WADA regulations, with infusions exceeding 100 mL per 12-hour period prohibited unless medically justified for diagnostic, surgical or therapeutic purposes. As such, IV delivery of supplements generally constitutes an anti-doping violation and is discouraged due to both regulatory constraints and associated risks. The primary exception occurs when medical intervention is necessary to safeguard an athlete’s health.

**Table 1.** Characteristics of ergogenic supplements used to directly enhance cycling performance.

Supplement	AIS group	Function	How it works	Typical dose	Evidence
Beta-alanine	A	Buffers lactic acid, delaying fatigue in high-intensity efforts	Increases muscle carnosine, which helps buffer hydrogen ions during intense exercise	3.2 to 6.4 g/day over 4 to 6 weeks	Shown to improve performance in high-intensity cycling efforts lasting 1–4 minutes, such as hill climbs or time trials
Caffeine	A	Enhances endurance, alertness, and perceived effort	Stimulates the central nervous system, reducing the perception of fatigue and enhancing focus.	3 to 6 mg/kg of body weight, taken about 60 minutes before exercise	Strong evidence supports its ability to improve time-trial performance and endurance in both trained and recreational cyclists
Carbohydrates	A	Fuels prolonged activity and delays fatigue	Maintains blood glucose and muscle glycogen levels during exercise	30 to 90 g/hour during exercise, depending on duration/intensity	Well-established benefits for all endurance activities; multiple transportable carbohydrates (glucose + fructose) enhance absorption
Carnitine	B	Enhances fat metabolism, may reduce lactate accumulation and support recovery	Facilitates transport of long-chain fatty acids into mitochondria for *β*-oxidation	2 to 3 g/day with carbohydrates, over ≥ 12 weeks	Chronic supplementation (with carbohydrate) increases muscle carnitine content and may reduce glycogen use and improve performance in prolonged endurance cycling. Benefits are context-specific and require long-term use.
Creatine monohydrate	A	Improves short bursts of high-intensity effort.	Increases phosphocreatine stores in muscles, aiding in rapid energy production	0.1 g/kg/day over 3 to 4 weeks	While more effective in sports with repeated sprint demands, it can benefit track cycling and sprint intervals
Dietary nitrates	A	Enhances endurance and reduces oxygen cost during submaximal exercise	Converts to nitric oxide in the body, promoting vasodilation and improved muscle efficiency	300 to 600 mg of nitrate (~500 ml beetroot juice), 2 to 3 hours pre-exercise	Proven benefits for endurance cyclists, especially in time trials and moderate-intensity efforts
Electrolytes	A	Support fluid balance, thermoregulation, and neuromuscular function during prolonged or intense exercise.	Help maintain plasma volume, nerve conduction, and muscle contraction. Sodium, the major ion lost in sweat, promotes fluid retention and supports cardiovascular stability.	300 to 700 mg of sodium per hour, adjusted based on individual sweat rate, exercise intensity, and environmental conditions.	Strong evidence supports the role of electrolyte supplementation in preventing dehydration-induced performance declines. Maintaining electrolyte balance helps preserve cardiovascular output, delay fatigue, and reduce the risk of cramping, particularly in hot or humid conditions. Individualised hydration strategies based on sweat testing are recommended for optimal outcomes.
Exogenous ketones	B	May improve endurance, cognitive function, and recovery during prolonged exercise by serving as an alternative energy substrate.	Increases circulating ketone bodies (primarily *β*-hydroxybutyrate), which can be used by skeletal muscle and the brain as an alternative to glucose or fatty acid	10 to 25 g of ketone monoester or ketone salts, 30 to 60 minutes before exercise.	Preliminary evidence shows mixed but promising effects on endurance performance, particularly under glycogen-depleted or fasted conditions. Benefits may be context-specific and influenced by co-ingestion with carbohydrates.
Glycerol	A	Improves hydration and thermoregulation in hot conditions	Increases total body water by promoting fluid retention, enhancing fluid availability during exercise and delaying dehydration	1.0 to 1.2 g/kg with 25 mL/kg fluids consumed 60 minutes before exercise	Supported for use in hot environments; improves hydration status and may improve endurance performance by reducing thermal and cardiovascular strain
Menthol	B	Improves thermal comfort and performance in hot conditions	Activates TRPM8 receptors in the mouth and upper airway, providing cooling sensation and improving perceived thermal comfort	0.01 to 0.05% menthol mouth rinse or beverage every 10 to 15 minutes during exercise in the heat	Growing evidence supports perceptual benefits and small improvements in endurance performance in hot conditions; more research needed for optimal dosing strategies
*N*-acetylcysteine	B	Antioxidant support, fatigue resistance during high oxidative stress	Serves as a precursor to glutathione, helping reduce oxidative stress and muscle fatigue	600 to 1200 mg/day for 5 to 9 days, sometimes acutely pre-exercise	Evidence shows NAC may improve performance or recovery during periods of high training load or oxidative stress, but results are inconsistent.
Quinine	B	May help reduce exercise-associated muscle cramps	Modulates motor neuron excitability by affecting sodium channels, potentially dampening hyperactive motor units	200 to 500 mg/day (quinine sulphate) in clinical contexts	Limited and context-specific. Some evidence from clinical populations for cramp reduction, but efficacy in athletes is unclear and safety concerns limit widespread use
Sodium bicarbonate	A	Buffers acid buildup during intense exercise, improving high-intensity performance.	Acts as an extracellular buffer, neutralising acid in the blood and delaying muscle fatigue	0.2 to 0.3 g/kg of body weight, taken 60 to 90 minutes before exercise (may cause gastrointestinal discomfort)	Effective for performance in high-intensity events lasting 1–10 minutes, such as short time trials or repeated sprint intervals

**Table 2.** Primary benefits, uses and risks associated with supplements to directly or indirectly enhance cycling performance.

Supplement	Primary effect	Most useful	Major risk(s)
Beta-alanine	Intracellular buffering	Short efforts (1 to 4 mins)	Paraesthesia (tingling sensation), potential gastrointestinal discomfort
Caffeine	Alertness, reduced fatigue	All cycling disciplines	Gastrointestinal discomfort, anxiety, sleep disruption, habituation (reduced effect over time)
Calcium	Bone health, neuromuscular	High training loads, athletes at risk of deficiency	Impaired absorption if combined with inhibitors, excessive intake may displace other minerals
Carbohydrates	Fuel supply, glycogen sparing	Endurance and time-trial	Gastrointestinal distress if poorly timed or overconsumed; rebound hypoglycaemia if intake is mistimed
Carnitine	Enhances fat metabolism, may reduce lactate accumulation	Aerobic efforts in low-glycogen states	Requires long-term use with high carbohydrate intake; potential gastrointestinal discomfort
Cherry juice	Anti-inflammatory, supports recovery	Recovery periods, post-race or training blocks	Gastrointestinal discomfort, potential allergic reaction to anthocyanins
Collagen	Supports tendon and ligament structure	Injury recovery and prevention phases	Limited evidence; contamination risk with low-quality products
Creatine	Sprint power, high-intensity bursts	Track, sprint, criterium	Water retention, potential weight gain, gastrointestinal discomfort
Curcumin	Anti-inflammatory, reduces muscle soreness and damage	Post-exercise recovery, high intensity training	Gastrointestinal discomfort at high doses, unknown long-term effects on training
Dietary nitrates	Improved oxygen efficiency	Submaximal endurance efforts	Gastrointestinal discomfort, potential drop in blood pressure (hypotension)
Electrolytes	Hydration, thermoregulation	Long rides, hot climates	Overconsumption may lead to hypernatremia or gastrointestinal discomfort
Exogenous ketones	Alternative energy substrate; potential glycogen sparing	Prolonged endurance efforts	Gastrointestinal distress (especially with ketone esters), taste and palatability, metabolic acidosis in rare cases
Glycerol	Cell hydration, thermoregulation	Hot environments, long-duration events	Overhydration (hyponatraemia) if not paired with sodium, gastrointestinal discomfort
Iron (if deficient)	Oxygen transport	Endurance performance	Iron overload (hemochromatosis), gastrointestinal irritation, constipation
Menthol	Cooling sensation, reduced perceived thermal strain	Hot conditions, prolonged exertion	Strong taste, may cause gastrointestinal discomfort or respiratory irritation
Multivitamin	General micronutrient support	Nutrition gaps or restricted diets	Low risk when taken appropriately, may mask deficiency symptoms
*N*-acetylcysteine	Antioxidant, supports recovery during oxidative stress	Intense training blocks or high fatigue	Gastrointestinal discomfort, nausea at high doses, mixed efficacy evidence
Omega-3 fatty acids	Anti-inflammatory, supports recovery and cardiovascular health	High training loads, recovery phases	Bleeding risk at high doses (rare), gastrointestinal discomfort, potential fishy aftertaste
Pickle Juice	May relieve muscle cramps	During or after cramping episode	Unpleasant taste, uncertain efficacy, short duration of effect
Probiotics	Support gastrointestinal and immune health	During high training loads or illness recovery	Mild bloating or discomfort when stating, strain specific effects
Protein	Recovery and adaptation	All training phases	Kidney stress in individuals with pre-existing kidney disease (no risk in healthy individuals), digestive discomfort at very high doses
Quinine	Reduced perception of effort, enhances neural activation	Sprint cycling, high effort intervals	Unpleasant taste
Sodium bicarbonate	Extracellular buffering	Sprint intervals, time-trials	Significant gastrointestinal distress (nausea, diarrhoea, bloating) if not properly dosed
Vitamin C	Antioxidant support, immune function	Cold exposure, illness risk periods	High doses may impair exercise adaptation, gastrointestinal discomfort
Vitamin D	Bone health, immune support	Low sunlight exposure, winter training	Excess intake can lead to toxicity, interfere with calcium absorption
Zinc	Immune function, antioxidant	Illness recovery, immune support	Copper deficiency with long-term high doses, nausea

### 4.1. Evidence for ergogenic supplements in cycling

The AIS framework provides broad guidance to support the safe and effective use of supplements that is agnostic to the sporting discipline. A structured synthesis of key recent meta-analyses and systematic reviews evaluating the level of evidence for AIS Group A and B supplements specifically with respect to cycling performance was performed. This analysis demonstrated consistent agreement with AIS framework classifications for all supplements with the exception of quinine where evidence was lacking (**Supplemental Table 1**). All Group A supplements consistently improve cycling performance, with the strongest evidence for beta-alanine, caffeine, carbohydrate and sodium bicarbonate. These compounds reliably enhance time trial outcomes, endurance capacity and high-intensity efforts across both trained and recreational populations. Creatine monohydrate and dietary nitrate also show benefits, particularly for short-duration, high-intensity surges and endurance performance in select contexts, respectively. In contrast, Group B supplements such as carnitine, menthol and *N*-acetylcysteine display mixed or context-dependent effects, with clearer utility in specific scenarios such as sprint efforts, hot environments or prolonged multistage events. Evidence for exogenous ketones remains inconsistent and generally very low, with little indication of meaningful ergogenic benefit in cycling.

### 4.2. Beta-alanine (Group A)

Beta-alanine is a non-essential amino acid that serves as the rate-limiting precursor to carnosine, a dipeptide (*β*-alanyl-L-histidine) that is highly concentrated in skeletal muscle [16–18]. Carnosine plays a critical role in intracellular pH buffering, particularly during high-intensity exercise by neutralising hydrogen ions (H⁺) produced through anaerobic glycolysis [19]. The accumulation of H⁺ is closely associated with muscle acidosis, which impairs enzyme (including PFK-1) function and muscle contractility, ultimately leading to fatigue.

Supplementation with beta-alanine has been shown to support chronic elevations in muscle carnosine content, typically by 40 to 80% over 4 to 6 weeks [17]. This increase in carnosine enhances the muscle’s ability to buffer intracellular acidosis, effectively delaying the onset of fatigue during bouts of intense anaerobic work. This is particularly relevant for cycling disciplines that involve efforts lasting 1 to 4 minutes, such as hill climbs, breakaways, pursuit races and short time trials, where acid-base disturbances significantly influence performance outcomes.

The standard dosing protocol involves ingesting 3.2 to 6.4 g/day of beta-alanine, often split into smaller doses throughout the day to mitigate paraesthesia (a tingling sensation), which is a common but transient and benign side effect that does not require intervention. Co-ingestion with a meal has been shown to enhance absorption and may further reduce the likelihood and severity of sensory side effects compared to ingestion on an empty stomach. Sustained-release formulations can also help reduce this sensory response while maintaining efficacy.

Scientific evidence strongly supports the ergogenic effect of beta-alanine in cycling events that are limited by acidosis, including repeated sprint performance, time to exhaustion during high-intensity efforts and power output during efforts with a duration of 60 to 240 seconds [19]. Meta-analyses and controlled trials consistently demonstrate performance improvements in tasks falling within this duration range, with the magnitude of benefit typically around 2 to 3%, though individual responses may vary [17,20].

### 4.3. Caffeine (Group A)

Caffeine is one of the most extensively studied and widely used ergogenic supplements in both competitive and recreational endurance cycling. Its primary benefits include enhanced endurance capacity, increased alertness and a reduction in perceived effort [21]. These effects collectively contribute to improvements in performance, particularly in time-trial and prolonged endurance cycling events.

Caffeine exerts its physiological effects mainly through antagonism of adenosine receptors in the central nervous system (CNS). By blocking the action of adenosine, a neuromodulator associated with the onset of fatigue and decreased arousal, caffeine promotes increased neuronal firing and the release of excitatory neurotransmitters such as dopamine and norepinephrine [22]. This results in increased alertness and reduced perception of fatigue, which are advantageous during prolonged and intense efforts such as sustained climbing, breakaways or final sprints in a cycling race. Additionally, caffeine may enhance motor unit recruitment, improve calcium handling in skeletal muscle and increase the mobilisation of intracellular fat stores, thereby potentially sparing muscle glycogen during submaximal efforts [21]. While the glycogen-sparing effect appears to be more modest than originally thought, it may still contribute to prolonged endurance under certain conditions.

The optimal dosage of caffeine for performance enhancement ranges between 3 and 9 mg/kg of body weight, typically ingested approximately 45 to 60 minutes before exercise depending on formulation [23,24], for example Durvitan versus Coffeinum *N*, to coincide with peak plasma concentrations. Co-ingestion with food may delay absorption, potentially extending the time to reach peak plasma concentration. Therefore, for quicker onset of effects, these supplements are often taken on an empty stomach. This moderate dose range is sufficient to elicit performance benefits while minimising potential side effects such as gastrointestinal distress, jitteriness or elevated heart rate, which may occur at higher intakes (>9 mg/kg) or with chronic moderate ingestion over consecutive days [21].

Meta-analyses and randomised controlled trials consistently demonstrate that caffeine ingestion improves time-trial performance, power output and endurance capacity in both trained athletes and recreational cyclists [24]. For instance, time to exhaustion and mean power output during cycling tests are significantly increased with caffeine compared to placebo. These effects are evident across a range of intensities and durations, from short 5-minute efforts to prolonged 60-minute time trials [25].

### 4.4. Carbohydrate (Group A)

Carbohydrates are the primary fuel source for endurance cycling, playing a central role in sustaining high intensity efforts and delaying the onset of fatigue. As a supplement, carbohydrate ingestion before, during and after exercise supports performance by maintaining blood glucose levels, preserving muscle glycogen stores and facilitating rapid recovery [26]. The importance of carbohydrates is especially pronounced in prolonged cycling events, where endogenous glycogen stores can become depleted, limiting performance and contributing to fatigue. During exercise, skeletal muscle relies on a combination of muscle glycogen and blood glucose to meet the energy demands of aerobic (oxidative phosphorylation) and anaerobic (glycolytic) metabolism [27]. When exercise exceeds 60 to 90 minutes in duration, the availability of these carbohydrate sources becomes critical [28]. Supplementing with exogenous carbohydrates helps to spare muscle glycogen and maintain CNS function to prevent declines in power output and mental focus. Studies have also shown that during short-duration (45 to 60 minutes), high-intensity efforts, where glucose or glycogen availability is not limiting, carbohydrate mouth rinsing may offer performance benefits through central mechanisms [29]. This effect is thought to occur by reducing perceived exertion and enhancing motor output via activation of brain regions involved in reward processing and motor control [29,30]. A recent meta-analysis demonstrated that carbohydrate mouth rinsing increases power output, but did not consistently improve time trial performance [31].

The recommended intake of carbohydrates during endurance cycling events ranges from 30 to 90 g/hr, with the optimal amount depending on intensity and duration. For efforts exceeding 2.5 hours, higher intakes of 90 to 120 g/hr can be achieved by using multiple transportable carbohydrates, typically a combination of glucose and fructose [32]. These simple carbohydrates use distinct intestinal transporters in sodium-glucose co-transporter 1 (SGLT1) for glucose, and glucose transporter type 5 (GLUT5) for fructose, allowing for greater absorption and oxidation rates (up to 1.5 g/min), and minimising gastrointestinal discomfort associated with high single-source carbohydrate intake [33].

Numerous studies and meta-analyses demonstrate significant improvements in time-trial performance, time to exhaustion and mean power output with carbohydrate ingestion, particularly when compared to placebo or water alone [34,35]. Recent evidence suggests that increasing carbohydrate intake to 120 g/hr during prolonged endurance events may further attenuate muscle damage and improve recovery relative to the current 90 g/hr guideline [32]. Additionally, carbohydrate intake during intermittent high intensity cycling events can help sustain repeated efforts by supporting both aerobic and anaerobic metabolism.Beyond the acute performance benefits, chronic carbohydrate availability modulates molecular and physiological responses to training. A recent meta-analysis showed that exercise performed with low carbohydrate availability increases the expression multiple regulators of metabolic function and, when glycogen is substantially depleted, enhances PGC-1α expression, a key regulator of mitochondrial biogenesis, providing a mechanistic basis for endurance adaptations with low-glycogen training [36]. However, when translated to applied performance outcomes, the evidence indicates largely neutral effects. Specifically, in a five-week intervention in trained cyclists, a periodised carbohydrate diet incorporating low carbohydrate availability sessions did not confer superior improvements in maximal lactate steady state (MLSS) or endurance performance compared to a consistently high carbohydrate intake [37]. Collectively, these findings suggest that while low-carbohydrate training strategies may influence molecular signalling, the translation to performance benefits likely depends on training status, duration of intervention and individual responsiveness.

### 4.5. Carnitine (Group B)

L-carnitine is a conditionally essential nutrient involved in long-chain fatty acid metabolism, acting as a cofactor in the carnitine palmitoyl transferase (CPT) system, which facilitates mitochondrial uptake of fatty acyl-CoA for *β*-oxidation [38,39]. By promoting fatty acid transport across the mitochondrial membrane, L-carnitine enables enhanced lipid utilisation during aerobic metabolism, potentially preserving muscle glycogen stores and delaying fatigue during prolonged endurance exercise [40]. While acute supplementation has limited efficacy due to poor skeletal muscle uptake, chronic co-ingestion with high carbohydrate intake has been shown to elevate muscle carnitine concentration over 24 weeks [41]. This upregulation of intramuscular carnitine has been associated with reduced glycolytic flux and increased fat oxidation at submaximal intensities, as well as improved performance during high intensity intervals in trained individuals [42]. These metabolic adaptations are particularly relevant for cyclists during long duration aerobic events, stages with mixed intensity efforts, or in scenarios involving carbohydrate restriction or glycogen depletion.

The typical dosing strategy involves 2 to 3 g/day of L-carnitine tartrate or L-carnitine-L-tartrate, taken in conjunction with 40 to 80 g of carbohydrate to stimulate insulin mediated muscle uptake. Importantly, meaningful increases in skeletal muscle carnitine require prolonged supplementation, over 12 to 24 weeks, alongside repeated high-carbohydrate co-ingestion, imposing a substantial time and dietary burden that may limit real-world feasibility for many athletes. Consequently, despite mechanistic rationale and some positive findings from controlled studies, the ergogenic potential of L-carnitine appears highly context-dependent, with variable responsiveness and limited applicability in short-term training or competition phases. At present, L-carnitine is not considered a a broadly effective supplement for cyclists, but may provide niche benefits under carefully controlled, long term nutritional strategies during high-volume training periods [42].

### 4.6. Creatine monohydrate (Group A)

Creatine monohydrate is among the most well studied and effective dietary supplements for enhancing high intensity exercise performance [43]. The primary benefit of creatine in energy metabolism lies in the rapid resynthesis of ATP via the phosphagen system, which is particularly relevant during short duration, maximal intensity efforts such as sprints, accelerations and high power track cycling events. Creatine is stored in skeletal muscle as phosphocreatine (PCr), which donates a phosphate group to ADP to rapidly regenerate ATP during maximal exertion [44]. Supplementation with creatine monohydrate significantly increases intramuscular PCr stores, enhancing the muscle's ability to produce ATP during short bursts of activity. This mechanism supports improved performance during repeated sprint bouts, peak power output, and reduced recovery time between efforts; attributes critical in disciplines such as track cycling, criterium racing and hill sprints.

Traditional creatine supplement protocols involve a loading phase of 20 g/day, typically divided into four 5 g doses, over 5 to 7 days, followed by a maintenance dose of 3 to 5 g/day to sustain elevated intramuscular PCr stores. This approach achieves a ~20 to 40% increase in total muscle creatine and PCr content, depending on individual baseline stores and muscle fibre type distribution. This protocol may be associated with transient gastrointestinal discomfort in some individuals [45]. A recent protocol recommend a single daily dose of 0.1 g/kg body weight over 3 to 4 weeks has been shown to achieve similar elevations in muscle creatine and PCr without the loading phase, offering a more gradual and potentially better-tolerated alternative for long-term supplement strategies [43]. The timing of creatine supplementation around exercise has gained attention as a potential factor in optimising muscle loading and performance, though no clear consensus exists. Emerging evidence comparing pre-, during- and post- exercise dosing protocols, favours post-exercise intake; however, methodological limitations and lacking mechanistic data prevent definitive conclusions [46].

Although traditionally associated with sports requiring explosive power, creatine has demonstrated efficacy in enhancing sprint capacity within endurance sports, including cycling. Several studies have demonstrated improvements in performance measures such as peak power output, time to fatigue and repeated sprint ability among cyclists after creatine supplementation [47]. Notably, these benefits are more pronounced during activities involving intermittent high intensity efforts superimposed on a background of aerobic work, a pattern characteristic of many competitive cycling events. One consideration is a modest increase in body mass (1 to 2 kg), largely due to water retention within muscle cells. While this may be a concern for climbing or weight sensitive events, the trade-off may be favourable in disciplines emphasising sprint power and anaerobic capacity. In addition to these direct ergogenic effects, creatine has also been shown to support recovery by attenuating markers of muscle damage, inflammation and oxidative stress, potentially facilitating improved recovery kinetics between training sessions [48,49].

### 4.7 Dietary nitrates (Group A)

Dietary nitrate, commonly consumed in the form of beetroot juice, has emerged as a promising ergogenic supplement for cyclists, although responses vary according to training status. The greatest performance benefits are consistently observed in recreational to moderately trained cyclists, whereas effects in elite athletes tend to be smaller and more variable, likely reflecting already optimised vascular, metabolic and mitochondrial function. Nitrate (NO₃⁻) is reduced in the body via the nitrate to nitrite to nitric oxide pathway, which operates independently of oxygen and provides an alternative to the classical L-arginine pathways for nitric oxide synthesis [13]. The end product, nitric oxide, is a potent signalling molecule that mediates vasodilation, enhances muscle blood flow, improves oxygen delivery and increases mitochondrial efficiency [50].

Once ingested, dietary nitrates are absorbed and concentrated in the salivary glands, where oral bacteria reduce it to nitrite (NO₂⁻). This nitrite is then further reduced to nitric oxide, particularly under hypoxic and acidic conditions; precisely the environment present during strenuous exercise. Nitric oxide's vasodilatory effects result in reduced vascular resistance and increased perfusion of working muscles, potentially improving oxygen and nutrient delivery during submaximal exercise. In addition to vascular effects, dietary nitrates also enhance muscle contractile efficiency by improving mitochondrial coupling and reducing the ATP cost of force production [51]. These adaptations translate to a lower oxygen cost at a given submaximal output, which is particularly beneficial during prolonged cycling efforts or time trials where energy efficiency and pacing are critical.

The effective dose of dietary nitrate reported in a recent meta-analysis of 76 studies typically ranged from 300 to 600 mg, typically delivered via 500 mL of concentrated beetroot juice, consumed 2 to 3 hours prior to exercise to allow for peak plasma nitrite concentrations [52]. Chronic supplementation strategies (over several days) have also been explored, though acute pre-exercise dosing remains the most common approach [50].

Evidence supports the efficacy of nitrates in enhancing endurance cycling performance, particularly in events involving continuous moderate-intensity efforts lasting 10 to 40 minutes. Improvements include increased time to exhaustion, enhanced power output and reduced oxygen consumption at submaximal workloads. These benefits are most pronounced in recreational to moderately trained athletes, though elite athletes may still experience smaller gains [53].

### 4.8. Electrolytes and hydration products (Group A)

Hydration and electrolyte management are fundamental components of performance nutrition for endurance athletes, particularly cyclists. During prolonged or high intensity cycling, especially in hot or humid conditions, significant fluid and electrolyte losses can occur through sweat, leading to dehydration, electrolyte imbalances, and thermoregulatory stress, all of which impair physical and cognitive performance. Supplementing with electrolyte rich hydration products supports the maintenance of fluid balance, cardiovascular stability, muscle function and thermal homoeostasis, thereby enhancing both performance and safety [54]. Electrolytes, primarily sodium, potassium, magnesium and chloride, play critical roles in fluid retention, neuromuscular conduction and acid–base regulation. Sodium is the most important electrolyte lost in sweat and is essential for maintaining plasma osmolality and stimulating thirst, thereby promoting fluid intake and retention [55]. Potassium contributes to intracellular fluid balance and muscle cell function, while magnesium and chloride support enzymatic activity and electrolyte balance [56].

Electrolyte supplementation during endurance cycling aims to mitigate fluid and sodium losses that impair thermoregulation and performance. Practical protocols typically recommend ingesting 300 to 700 mg of sodium per hour, paired with fluids consumed at a rate aligned to individual sweat loss. However, actual sodium loss can vary substantially depending on environmental conditions, particularly heat and humidity, and individual physiology, with some athletes losing as much as 1,500 to 2,000 mg of sodium per hour. Therefore, personalised hydration strategies based on sweat testing or validated field assessments are increasingly used to tailor sodium and fluid intake to specific needs. While body mass losses of ~2% have often been cited as a threshold beyond which aerobic performance and thermoregulation may be impaired [57], recent work highlights that this relationship is more complex. Performance decrements depend on factors such as exercise intensity, environmental conditions, individual acclimation status and whether hydration is guided by thirst or programmed strategies [58,59]. Thus, although minimising dehydration is a reasonable goal, not all cases of >2% body mass loss uniformly result in significant thermoregulatory or performance decline.

Electrolytes can be consumed via sports drinks, hydration tablets or electrolyte powders, many of which are formulated with glucose to facilitate sodium–glucose co-transport via SGLT transporters in the gut. This co-transport mechanism enhances intestinal fluid absorption while simultaneously supporting carbohydrate availability, providing complementary benefits during prolonged cycling efforts. Numerous studies have demonstrated that adequate hydration, especially when paired with electrolytes, maintains cardiac output, sweat rate and skin blood flow, helping to dissipate heat and sustain power output during long-duration exercise [54]. Inadequate electrolyte intake, particularly sodium, has been linked to exercise associated muscle cramps and, in severe cases, hyponatremia when fluid intake is excessive and sodium is not replaced. Similarly, overconsumption of plain water without sodium replacement can predispose to hyponatremia in endurance cycling events.

### 4.9 Exogenous ketones (Group B)

Exogenous ketones, primarily available as ketone esters or ketone salts, are designed to elevate circulating ketone body concentrations, particularly *β*-hydroxybutyrate (*β*-HB), without the need for prolonged fasting or adherence to a ketogenic diet. Upon ingestion, these supplements rapidly increase plasma *β*-HB levels to between 1 and 3 mmol/L within 30 to 60 minutes, offering an alternative oxidative substrate for mitochondrial ATP production during endurance exercise [60]. Although early studies suggested theoretical advantages such as a higher ATP yield per unit of oxygen compared to glucose, glycogen-sparing effects, and attenuation of central fatigue through preserved glucose and neurotransmitter homoeostasis, more recent evidence has been inconsistent and does not robustly support these mechanisms under typical sporting conditions [61]. Performance benefits appear to be highly context-dependent, influenced by variables such as exercise intensity, duration, and carbohydrate availability [62].

In practice, the most promising application of exogenous ketones appears to lie in their potential to support recovery [63,64]. Post-exercise ingestion of ketone esters has been associated with enhanced glycogen resynthesis, attenuation of exercise-induced inflammation, and modulation of post-exercise hormonal responses that may favour an anabolic environment. These recovery-oriented effects, particularly when combined with carbohydrates and protein, have led to growing interest in their use between closely spaced training sessions or competitions.

A recent meta-analysis of 10 studies (8 cycling specific) showed that ketone esters are typically consumed in doses of 10 to 25 grams approximately 30 to 60 minutes before or after exercise [65]. While more palatable, ketone salts deliver lower *β*-HB concentrations and introduce a higher sodium load, potentially limiting their effectiveness. Gastrointestinal distress remains a common side effect, particularly with ester forms. Although the mechanistic rationale remains intriguing, further high-quality studies are needed to clarify the role of exogenous ketones in recovery and to determine optimal dosing protocols and practical utility in cycling.

### 4.10. Glycerol (Group A)

Glycerol is a three-carbon alcohol that has established efficacy in promoting hyperhydration and improving thermoregulatory capacity during prolonged or hot-weather cycling events. When co-ingested with large volumes of fluid, glycerol enhances fluid retention by increasing total body water through osmotic expansion of both intracellular and extracellular compartments [66]. This hyperhydration effect can delay dehydration, lower core temperature rise, and improve cardiovascular stability during prolonged endurance events. Glycerol is distributed throughout body water compartments, where it increases plasma osmolality and promotes fluid retention via reduced urinary output. Unlike electrolytes, which primarily influence extracellular fluid balance, glycerol’s osmotic properties facilitate expansion of intracellular fluid volume as well, resulting in a more sustained hydration state [67]. These effects may be particularly advantageous in endurance cycling disciplines, where fluid losses from prolonged sweating can impair performance and increase the risk of heat-related illness.

The typical dosing protocol involves ingesting 1.0 to 1.2 g/kg body weight of glycerol along with 25 to 35 mL/kg body weight of water 60 to 120 minutes prior to exercise [68]. This strategy has been shown to increase total body water by up to 700 mL, with corresponding improvements in exercise capacity in hotter climates. In addition to thermoregulatory benefits, glycerol supplementation has also been associated with improved time-trial performance and reduced perceived exertion under hot and humid conditions [66].

Glycerol was temporarily placed on the WADA prohibited list due to concerns about plasma volume expansion as a potential masking agent, it was removed in 2018 following re-evaluation of its use in sport. It remains approved for use under current anti-doping regulations, provided that dosing protocols remain within physiologically accepted limits. Minor side effects may include bloating, gastrointestinal discomfort and headaches in some individuals, particularly when large fluid volumes are ingested quickly.

### 4.11. Menthol (Group B)

Menthol has primarily been investigated for its perceptual cooling properties during exercise in hot environments. Menthol activates transient receptor potential melastatin-8 (TRPM8) receptors located in the oropharyngeal and skin regions, creating a sensation of cooling without directly altering core or skin temperature. This sensory illusion can reduce thermal discomfort, improve exercise tolerance and enhance performance in the heat, particularly during endurance cycling events.

When used in the form of mouth rinses, sprays or topical applications, menthol stimulates cold-sensitive neurons, leading to a subjective perception of reduced heat stress. While it does not affect thermoregulatory physiology, this perceptual modulation may enable athletes to maintain a higher work rate or delay voluntary exhaustion during prolonged efforts in hot conditions [69]. Typical application strategies involve menthol mouth rinsing with a solution containing 0.01 to 0.1% menthol, administered intermittently throughout exercise or during rest intervals. Studies have shown improved time-trial performance and reduced ratings of perceived exertion (RPE) during prolonged cycling in the heat with menthol rinsing compared to placebo [70,71]. The effects are most pronounced in settings of heat stress or where internal cooling strategies such as cold fluids or ice slurries are impractical.

Despite promising findings, menthol’s classification in Group B reflects the need for further research to confirm optimal dosing, timing and efficacy across various environmental contexts. Care must also be taken when using menthol in excessively hot environments, as perceptual cooling without corresponding physiological cooling could increase the risk of heat-related illness if it masks early symptoms of hyperthermia [72].

### 4.12. N-acetylcysteine (Group B)

N*-*acetylcysteine (NAC) is a precursor to the amino acid cysteine and serves as a key substrate for the synthesis of glutathione, the body’s principal intracellular antioxidant. In the context of endurance sports such as cycling, NAC supplementation is proposed to enhance performance and recovery by mitigating oxidative stress, preserving redox balance, and delaying fatigue during periods of high metabolic demand [73]. Exercise induced oxidative stress is especially pronounced during prolonged training blocks and multi-stage races, where accumulation of reactive oxygen species (ROS) can impair mitochondrial function, promote muscle damage, and reduce force output. By elevating intracellular glutathione levels, NAC supports endogenous antioxidant capacity and stabilises muscle redox status [74]. This may attenuate oxidative damage to contractile proteins and improve calcium handling, thus sustaining muscle function during extended efforts. In addition, NAC has been reported to modulate the central nervous system via redox-sensitive neurotransmission, potentially reducing central fatigue [75].

Typical supplementation protocols involve 600 to 1200 mg/day, taken orally for 5 to 9 days or acutely 60 to 90 minutes before and after exercise [76]. Daily supplementation before and after each stage in multiple stage races such as the Tour de France is routine practice. While NAC appears most effective during states of elevated oxidative stress such as training overload or heat stress, its ergogenic potential varies widely depending on dose, timing, and individual redox status. Some studies have demonstrated improved cycling time to exhaustion, reduced perceived exertion and enhanced recovery markers following NAC supplementation, particularly after intense training [77]. However, others report no benefit, reflecting inter-individual variability and potential ceiling effects in trained athletes with already robust antioxidant systems [78,79].

Although not as widely adopted as more established ergogenic supplements, NAC may be especially useful to shorten the recovery period for endurance cyclists during periods of competition, travel or heat exposure; conditions known to exacerbate oxidative stress and impair recovery. Additionally, emerging evidence suggests that NAC may complement nutritional strategies aimed at preserving mitochondrial function and managing training induced fatigue, making it a potentially valuable component of targeted supplementation plans. When considering the use of NAC during intense training blocks, the benefits in terms of shortened recovery need to be balanced against the potential to impede training adaptation. Accordingly, chronic or indiscriminate use of NAC during key training phases should be approached with caution, as prolonged suppression of exercise-induced oxidative stress may blunt redox-sensitive signalling pathways involved in mitochondrial biogenesis and endurance adaptation [80].

### 4.13. Quinine (Group B)

Quinine is a bitter alkaloid extracted from the cinchona tree and has historically used as an antimalarial agent. Its proposed application as an ergogenic aid is based on its ability to activate bitter taste receptors in the oral cavity and upper gastrointestinal tract, which may enhance corticomotor excitability and influence central perception of effort during high-intensity exercise [81,82]. However, this proposed mechanism remains speculative and is supported by a very limited number of experimental studies.

Unlike carbohydrate mouth rinsing, quinine must be ingested to elicit physiological effects, as the relevant receptors are located in the back of the throat and gut [83]. A small number of studies in trained cyclists have reported modest improvements in short duration, high intensity performance, for example a 2.5 to 4% greater output during 30-second sprints and enhanced early-stage performance in 3 to 4-minute cycling efforts [81]. These findings are not universal, are based on small sample sizes, and appear to reflect centrally mediated effects on pacing or neural drive rather than robust metabolic or physiological adaptations.

Accordingly, the current evidence base supporting quinine as an ergogenic aid is weak. Quinine’s classification in Group B reflects preliminary interest rather than established efficacy. Practical application is further limited by its strong bitter taste, potential gastrointestinal discomfort and rare but serious adverse effects at higher doses includingcardiac issues (arrhythmia). Until larger, well-controlled studies demonstrate consistent performance benefits and clarify safety, quinine should be considered experimental and used with caution, if at all, in athletic settings [84].

### 4.14. Sodium bicarbonate (Group A)

Sodium bicarbonate (NaHCO₃) is a well-established extracellular buffering agent used to enhance performance in high intensity exercise by mitigating the effects of exercise induced acidosis [85]. During high intensity efforts lasting between 1 and 10 minutes, such as short time trials, breakaway surges or repeated sprint intervals, the reliance on anaerobic glycolysis leads to the accumulation of hydrogen ions (H⁺) and a subsequent decline in intracellular and extracellular pH [86]. This acidification impairs muscle contractility, enzyme activity and energy production, contributing to early fatigue.

Sodium bicarbonate supplementation increases blood bicarbonate concentration and stabilises pH, enhancing the body’s capacity to buffer and remove H⁺ from working muscles into the circulation. This augmented proton gradient facilitates greater efflux of H⁺ from the intracellular space, thereby delaying intracellular acidosis and maintaining a more favourable pH environment for ATP production and muscle contraction.

The typical dosing strategy involves ingesting 0.2 to 0.3 g/kg body weight of sodium bicarbonate approximately 60 to 90 minutes prior to exercise, allowing time for peak buffering effects to occur. Despite its effectiveness, a common limitation of sodium bicarbonate use is the risk of gastrointestinal distress, including bloating, nausea and diarrhoea [87]. Additionally, since a standard 21 g dose of sodium bicarbonate (used for a 70 kg athlete) contains approximately 5.75 g of sodium, individuals who are sodium-sensitive may experience weight gain due to fluid retention. Strategies to minimise discomfort include divided dosing, co-ingestion with a meal, use of enteric-coated capsules or chronic loading protocols spread over multiple days [85].

The ergogenic effects of sodium bicarbonate are most evident in high intensity, short duration efforts where metabolic acidosis is a performance limiting factor. Cycling studies have demonstrated significant improvements in mean and peak power output, repeated sprint performance and time to exhaustion following bicarbonate ingestion [88]. This makes sodium bicarbonate particularly useful in disciplines such as track cycling, criteriums or stages with frequent changes in pace.

## 5. Synergy and redundancy in ergogenic supplement regimens

In practice ergogenic supplements are rarely used in isolation. Athletes often adopt multi supplement regimens to address multiple physiological demands such as energy availability, fatigue resistance, hydration and recovery. Understanding potential synergies and redundancies between supplements is crucial for optimising performance outcomes and avoiding ineffective or overlapping strategies. Documented and proposed supplement synergies and redundancies in cycling are detailed in Table 3.

**Table 3.** Documented and proposed supplement synergies and redundancies in cycling.

Supplement combination	Classification	Primary mechanism(s)	Evidence summary	Practical notes
Beta-alanine + Sodium bicarbonate	Synergistic	Intracellular + extracellular buffering	Additive performance benefits in high-intensity efforts (1–4 min)	Increased GI risk; requires dose titration
Caffeine + Carbohydrate	Synergistic	Central stimulation + substrate availability	Consistent improvements in time-trial performance and TTE	Widely used; individual caffeine tolerance important
Creatine + Beta-alanine	Potential synergy	PCr resynthesis + buffering	Modest benefits, stronger in resistance-trained athletes	Limited relevance for endurance cycling
L-carnitine + Carbohydrate (chronic)	Conditional synergy	Insulin-mediated carnitine uptake	Improved fat oxidation after long-term use	High time and dietary burden
Carbohydrate + Electrolytes	Synergistic	SGLT-mediated absorption, hydration + fuel	Strong evidence in prolonged endurance events	Core strategy, low risk
Carbohydrate + Ketones	Potentially redundant	Competing oxidative substrates	Ketone oxidation may be blunted with high CHO intake	Context dependent
Multiple buffering agents (e.g. BA + NaHCO₃ + ketones)	Redundant	Overlapping alkalosis	No clear additive benefit	Increased GI distress risk
NAC + other antioxidants	Potentially redundant	Redox modulation	Mixed outcomes; adaptation concerns	Use with caution during training

Among the best-documented synergies is the combination of beta-alanine and sodium bicarbonate, which target complementary aspects of acid–base balance. Beta-alanine enhances intracellular buffering via elevated carnosine, while sodium bicarbonate improves extracellular buffering. Together, these supplements may optimise both intracellular and extracellular pH regulation, further enhancing tolerance to high-intensity workloads [89,90]. Meta-analyses support the additive benefit of this combination for high-intensity efforts, particularly in repeated sprints or time trials of 1 to 4 minutes duration [91]. Caffeine and carbohydrates are another well supported pairing. Caffeine acts centrally to reduce perceived exertion and may enhance carbohydrate oxidation during endurance exercise. When combined, these supplements consistently improve time-trial performance and time to exhaustion to a greater extent than the sum of either supplement alone [92]. Their synergy likely arises from overlapping central and peripheral effects whereby caffeine improves vigilance and neuromuscular recruitment, while carbohydrates maintain substrate availability and delay fatigue. Creatine and beta-alanine have also been investigated for their combined effects on strength and repeated sprint performance. While they act through distinct mechanisms in supporting ATP resynthesis and pH buffering, respectively, some trials report modest synergistic benefits, though these findings are more consistent in resistance trained athletes rather than in cyclists [93]. The interaction between creatine and beta-alanine appears non-redundant but may be context dependent. L-carnitine and carbohydrates may act synergistically during chronic supplementation, as insulin-mediated uptake is critical for increasing muscle carnitine content. Studies have shown improved fat oxidation and performance following co-ingestion over several weeks [41]. However, acute performance benefits remain inconsistent, and this strategy is best suited to athletes engaging in long-duration, submaximal cycling.

In contrast, potential redundancies arise when supplements target similar mechanisms. For example, exogenous ketones and carbohydrates may compete as fuel substrates. While ketones can spare glycogen and provide oxidative energy, co-ingestion with high carbohydrate loads may blunt ketone uptake and oxidation, possibly diminishing their theoretical benefit [94]. Similarly, combining multiple buffering agents such as beta-alanine and sodium bicarbonate with high dose exogenous ketones, which also induce mild alkalosis, may not produce additive benefits and may exacerbate gastrointestinal side effects. N-acetylcysteine has shown mixed results when combined with other recovery-focused interventions. While NAC may attenuate oxidative damage during heavy training or heat stress, its effects are variable and may interact with other antioxidant strategies. Importantly, chronic high-dose antioxidant use including NAC has been suggested to blunt exercise-induced mitochondrial adaptations, raising concerns about long-term use during training [95].

While combining supplements to target multiple physiological systems is common practice, increasing stacking complexity also elevates the risk of gastrointestinal distress, poor adherence, and unintended interactions. High total solute loads, overlapping buffering agents, or concurrent ingestion of multiple osmotically active compounds (e.g. bicarbonate, ketones, carbohydrates) may exacerbate GI symptoms and negate potential performance benefits. Accordingly, supplement stacking should be individualised, evidence-informed, and trialled during training rather than competition, with priority given to combinations supported by robust data and clear mechanistic complementarity. Beyond these documented synergies and redundancies between supplements that have been studied and have empirical evidence, there are a number of additional potential synergies and redundancies based on the physiological mechanism of these compounds that have not currently been resolved.

## 6. Medical supplements to indirectly support cycling performance

In addition to direct acting ergogenic supplements, several WADA permitted supplements indirectly enhance cycling performance by supporting recovery, physiological resilience and overall health (Table 2). Though not directly performance enhancing in the acute sense, these supplements contribute to long term performance sustainability and are valuable components of comprehensive nutrition strategies.

### 6.1. Calcium (Group A) for bone and neuromuscular health

Calcium is the most abundant mineral in the human body and plays vital roles in muscle contraction, nerve transmission, vascular function, hormone secretion and maintaining plasma calcium homoeostasis. For athletes, adequate calcium intake is critical not only for bone health but also for neuromuscular function during training and competition [96].

While calcium requirements for athletes do not exceed those of the general population, certain factors including low energy availability, vitamin D insufficiency, menstrual disturbances and sweat-induced dermal calcium losses, may increase calcium demands or compromise calcium balance. Studies have shown that acute calcium loss during exercise, particularly in hot and humid conditions, can stimulate parathyroid hormone (PTH) secretion and increase bone resorption. Consuming a calcium-rich meal (≥1000 mg) before or during exercise may mitigate these effects and help preserve bone health [97]. Athletes at risk of suboptimal calcium intake include those with restricted energy intake, dairy-free or vegan diets, malabsorption syndromes such as coeliac disease, or those following elimination diets low in bioavailable calcium [98]. Low calcium status is also more prevalent in athletes with menstrual dysfunction, high-protein plant-based diets or disordered eating patterns. In these populations, the risk of stress fractures and long-term osteoporosis is elevated.

Standard recommended daily intake of calcium ranges from 1000 to 1300 mg/day depending on age and sex. While food-based sources such as dairy, fortified plant milks and leafy greens are preferred due to their accompanying micronutrient content, use of calcium supplements may be warranted in cases of osteopenia/osteoporosis or persistently low dietary intake. Calcium carbonate is commonly used in supplements, with recommended doses of 500 to 600 mg of elemental calcium per serving to avoid reduced absorption. Calcium is a foundational nutrient in skeletal development, injury prevention and maintaining musculoskeletal function. Its role is particularly relevant in endurance athletes and others at risk for low energy availability or stress-related bone injuries.

### 6.2. Cherry juice (Group B) for recovery

Tart cherry juice, derived from Montmorency cherries, has gained interest as a supplement primarily based on the potential to reduce exercise-induced muscle damage (EIMD), inflammation and oxidative stress. These effects are attributed to high concentrations of polyphenolic compounds, particularly anthocyanins, which exhibit strong antioxidant and anti-inflammatory properties [99,100]. While not traditionally considered a direct ergogenic supplement, tart cherry juice may enhance performance indirectly by supporting recovery and maintaining neuromuscular function between training sessions or competitive events. The proposed mechanism involves attenuation of the secondary muscle damage response by reducing ROS production and modulating inflammatory cytokines such as IL-6 and TNF-*α*. In endurance and intermittent cycling events, repeated mechanical strain can compromise muscle integrity and function, leading to performance decrements in subsequent efforts. By dampening this response, tart cherry juice may preserve force production and reduce perceptions of soreness and fatigue.

Typical supplementation protocols involve consuming 30 to 60 mL of tart cherry concentrate or 240 to 480 mL of juice (equivalent to 600 mg polyphenols) twice daily for 4 to 7 days prior to a strenuous event, with continued use for 2 to 3 days following the event to support recovery [101]. While generally well tolerated, high polyphenol intake may interfere with certain cellular adaptations to training if used chronically, and thus its use is best reserved for competition phases or high training loads. Studies in endurance sports have demonstrated that tart cherry juice can improve recovery of strength and accelerate return to baseline performance following muscle-damaging exercise [102]. Tart cherry juice has been associated with reduced markers of oxidative stress, faster recovery of maximal voluntary contraction and improved performance in time trials conducted within 48 hours of fatiguing protocols [103]. Although more evidence is needed to classify tart cherry juice as a direct performance enhancer, its recovery benefits make it a promising adjunct to a comprehensive nutrition strategy for high-performance athletes.

### 6.3. Collagen support (Group B) for tissue repair

Collagen is the most abundant structural protein in the human body, primarily located within the extracellular matrix of connective tissues. Type I collagen, the dominant form skeletal muscle, is rich in the non-essential amino acids glycine, proline and hydroxyproline, which form a rigid triple-helical structure crucial for tensile strength and tissue integrity [104]. Collagen’s role in tissues involved in movement has made it a focus of interest for athletes seeking to support joint, tendon and ligament health, particularly in the context of injury prevention or rehabilitation.

Emerging research suggests that collagen synthesis is enhanced when collagen peptides are consumed in proximity to exercise, which acts as a potent stimulus to “switch on” the synthetic machinery of poorly vascularises tissues such as tendons [105]. For example, when vitamin C enriched gelatine is consumed one hour before exercise, circulating markers of collagen synthesis and amino acid availability are elevated, potentially enhancing connective tissue repair and resilience [106]. Sufficient or supplemented vitamin C is required as it serves as a co-factor in collagen synthesis pathways. Beyond structural support, glycine, which is the most abundant amino acid in collagen, may exert anti-inflammatory effects and support connective tissue recovery during periods of heightened mechanical stress or inflammation [107]. This has particular relevance for endurance athletes, who may experience chronic joint loading, micro-injuries, or overuse conditions such as tendinopathies.

Typical dosing protocols involve 10 to 15 grams of hydrolysed collagen or gelatine taken approximately one hour before loading exercise, often with 50 mg of vitamin C to support collagen formation [106]. While preliminary findings are promising, more research is needed to determine optimal dosing strategies, timing and long-term efficacy across athlete populations and injury types. Although collagen supplementation does not enhance endurance capacity or power output, its potential to support tissue repair, reduce exercise induced joint pain and maintain musculoskeletal health makes it a potentially valuable adjunct to athlete recovery and injury prevention programs.

### 6.4. Curcumin (Group B) for recovery and muscle soreness

Curcumin supplementation does not directly improve acute cycling performance but may support recovery and reduce muscle soreness following intense or prolonged exercise. Curcumin, a polyphenolic compound derived from turmeric, has demonstrated anti-inflammatory and antioxidant properties that may help attenuate EIMD, delayed onset muscle soreness (DOMS), and oxidative stress [108,109]. These effects are thought to arise from curcumin’s ability to modulate inflammatory pathways (e.g. COX-2, NF-κB) and reduce circulating markers of muscle damage such as creatine kinase (CK) [109].

Effective curcumin dosing strategies typically involve 500 to 2,500 mg taken once or twice daily for several days before and after strenuous exercise. Enhanced formulations, often containing piperine or other bioavailability enhancers, are recommended due to the poor systemic absorption of native curcumin. Supplementation has been associated with improved muscle recovery, reduced soreness, and maintenance of power and performance following eccentric or plyometric training loads [108].

Evidence supports the use of curcumin in reducing muscle damage markers, accelerating recovery, and alleviating soreness, particularly in the context of high-load or high-volume training. While it does not enhance endurance performance directly, curcumin supplementation may indirectly benefit cyclists by improving recovery kinetics, allowing for greater training consistency and adaptation over time. However, further high-quality research in sport-specific contexts is required to define optimal dosing and long-term impact on performance outcomes.

### 6.5. Iron (Group A) for iron-deficient cyclists

Iron is an essential micronutrient that supports oxygen transport and utilisation through its role in haemoglobin, myoglobin and various oxidative enzymes. In endurance sports like cycling, where oxygen delivery to working muscles is a key determinant of performance, iron status is critically important. However, iron supplementation should only be undertaken when iron deficiency is diagnosed, as excess iron can lead to oxidative stress and other health complications [110].

Iron deficiency, which is common among female athletes, vegetarians and those undergoing high training volumes, can impair oxygen-carrying capacity, reduce maximal oxygen uptake (VO_2_max), and lead to symptoms such as fatigue, decreased power output and slower recovery [110]. In cyclists with iron deficiency, supplementation restores haemoglobin concentration and oxygen transport, thereby augmenting mitochondrial respiration. Diagnosis typically involves assessment of serum ferritin, haemoglobin and transferrin saturation. When deficiency is confirmed, oral iron supplements such as ferrous sulphate (100 to 200 mg/day) can replenish iron stores and reverse performance deficits. Absorption of oral iron supplements is increased by avoiding co-ingestion with caffeine and calcium (dairy products).

Evidence supports improved aerobic capacity and training quality in athletes with iron deficiency following supplementation [111]. However, in iron-replete individuals, additional supplementation offers no direct or indirect benefit. Thus, iron is best viewed as a performance preserving rather than performance enhancing supplement, essential for maintaining optimal physiological function and avoiding performance decline due to anaemia or subclinical deficiency.

Consensus statements from the International Olympic Committee (IOC) and the American College of Sports Medicine (ACSM) recommend regular screening of iron status in endurance athletes, particularly females, adolescents and athletes at risk of low energy availability (LEA) or relative energy deficiency in sport (RED-S) [112]. Ferritin thresholds for intervention have been updated, with values <30 µg/L in men and <50 µg/L in women commonly used as indicators of depleted iron stores in athletes [113,114]. Routine monitoring is encouraged during heavy training, altitude exposure or when unexplained fatigue is present. In cases of functional iron deficiency or iron deficiency without anaemia (IDNA), supplementation may still be warranted to preserve training quality and reduce risk of progression to anaemia. Intravenous (IV) iron is reserved for clinically diagnosed, refractory cases where oral supplementation is poorly tolerated or ineffective and should only be administered under medical supervision due to anti-doping regulations and potential risks. Iron deficiency in the context of RED-S may compound impairments in endurance performance and recovery [112], highlighting the need for early detection and coordinated management involving sports physicians, dietitians and performance staff.

Importantly, these interventions are relevant not only for enhancing cycling performance but also for supporting athlete health. For instance, iron supplementation is commonly used in preparation for altitude exposure to build iron stores and reduce the risk of altitude-induced anaemia. Similarly, blood biomarkers such as elevated mean corpuscular volume (MCV) may guide the targeted use of B-vitamin complexes to prevent progression to megaloblastic anaemia.

### 6.6. Multivitamins (Group B) for general health

Multivitamin supplements combine essential micronutrients in a compact format and are used by athletes aiming to support general health, fill dietary gaps or cope with increased physiological demands. Vitamins and minerals in general play crucial roles in energy metabolism, immune defence, tissue repair, red blood cell production and bone health [115]. However, there is no robust evidence that multivitamin supplements support performance in well-nourished individuals, and their use should primarily be targeted at correcting or preventing diagnosed deficiencies.

Athletes at risk of suboptimal micronutrient intake include those with restricted energy availability, limited food variety (e.g. vegetarian diets) or high travel demands. In these populations, multivitamins may support nutrient replenishment and serve as a practical adjunct where food intake is insufficient. Specific micronutrients found in multivitamins including antioxidants, iron, vitamin B and vitamin D do contribute to physiological processes relevant to training adaptation, immune protection and recovery. These benefits are typically observed with isolated supplementation at doses exceeding those found in standard multivitamins and are considered separately in this review.

Despite their convenience, multivitamins are not a substitute for a nutrient-rich diet and present several limitations. Their broad formulation may not address individual deficiencies effectively and can lead to nutrient-nutrient interactions, excessive accumulation or adverse effects, which can be as serious as peripheral neuropathy from excessive vitamin B6 intake. Overreliance on multivitamins may also detract from consumption of whole foods rich in phytochemicals not captured in supplement form [116]. Multivitamins may be considered in athletes with identified micronutrient insufficiencies, poor dietary patterns or during periods of increased stress or travel. However, their use should be guided by clinical assessment and employed as an adjunct, not a replacement for, targeted nutritional strategies and professional dietary advice.

### 6.7. Omega-3 fatty acids (Group B) for health and resilience

Omega-3 fatty acids, primarily eicosapentaenoic acid (EPA) and docosahexaenoic acid (DHA), are long-chain polyunsaturated fats that exert broad systemic effects through their anti-inflammatory, membrane-stabilising and cardioprotective properties. In the context of endurance cycling, omega-3s do not directly enhance power output or VO₂max but contribute to training capacity and recovery by modulating inflammatory responses, promoting vascular function, and supporting immune health [117].

Fatty acids are incorporated into cell membranes, where they influence fluidity, receptor function and the production of eicosanoids, which are bioactive compounds involved in inflammatory signalling. Supplementation has been associated with reductions in DOMS, creatine kinase (CK) levels and markers of oxidative stress following intense exercise. Omega-3s may also enhance endothelial function and red blood cell deformability, potentially supporting oxygen delivery and aerobic efficiency in endurance athletes [118].

Typical dosing ranges from 1 to 3 g/day of combined EPA and DHA, with fish oil and algal oil being the most common sources. Chronic supplementation (≥4 weeks) appears necessary to realise full physiological benefits [119]. While not classified as direct ergogenic supplement, omega-3 fatty acids contribute meaningfully to athlete health, reduce training disruption, and may optimise adaptation through improved recovery kinetics.

### 6.8. Pickle juice (Group B) to mitigate cramping

Pickle juice, typically composed of vinegar, water, salt, and spices, has been investigated primarily for its role in mitigating exercise-associated muscle cramps (EAMCs). Unlike electrolyte-rich sports drinks, pickle juice appears to exert its effects not through hydration or electrolyte replacement but via a rapid, neural mechanism that inhibits cramp onset or reduces cramp duration [120].

The primary hypothesis is that compounds in pickle juice, particularly acetic acid and other pungent constituents, stimulate oropharyngeal and gastrointestinal TRP (transient receptor potential) ion channels. This sensory stimulation is believed to activate afferent neurons that influence spinal motor neuron excitability, thereby inhibiting the alpha-motor neurons responsible for sustained involuntary muscle contractions. This neuromodulatory response occurs rapidly, often within 30 to 90 seconds, and has been shown to shorten cramp duration without affecting systemic electrolyte or hydration status [121].

Typical dosing involves ingestion of 60 to 80 mL of pickle juice at the onset of cramping. While there is no universally established protocol, this volume is based on the quantities used in clinical trials. Due to its high sodium content and acidity, excessive or routine consumption is discouraged, especially for individuals with hypertension or gastrointestinal sensitivity. It is not recommended as a general hydration or electrolyte solution, as it lacks the balanced composition of targeted rehydration products. Despite the limited scope of action, pickle juice may be a valuable addition in specific athletic contexts, particularly in endurance or high-heat events where athletes are prone to cramping despite adequate hydration. Further research is needed to better define the mechanistic, safety and possible interaction profiles with other supplements.

### 6.9. Probiotics (Group B) for intestinal and immune support

Probiotic supplements contain live microorganisms, primarily from the genera *Lactobacillus*, Bifidobacterium, *Streptococcus* and *Saccharomyces*, that may support health benefits when consumed in adequate amounts. Their primary use among athletes is to support gastrointestinal and immune function, particularly during periods of heavy training or travel that challenge systemic resilience.

Probiotics may exert their effects by increasing the abundance of beneficial gut bacteria, enhancing short-chain fatty acid (SCFA) production, supporting gut barrier integrity and modulating local and systemic immune responses. In athletes, this could translate to improved tolerance to exercise-induced gastrointestinal syndrome (EIGS) and a reduction in upper respiratory symptoms (URS). However, systematic reviews report inconsistent effects on GI integrity, microbial diversity or SCFA concentrations, and there is currently no clear evidence of translation into improved exercise performance [122,123].

Studies in cycling have demonstrated that probiotic supplementation may reduce the incidence and severity of URS during intensive training and stage racing, which can indirectly support performance continuity. For example, *Lactobacillus fermentum* VRI-003 reduced the duration and severity of respiratory illness in competitive cyclists over an 11-week winter training block, particularly in male athletes [124]. Other studies have reported improved maintenance of training load and reduced gastrointestinal symptoms during simulated competition when probiotics were administered for 4–12 weeks [125]. There is no evidence of direct improvements in time trial performance or power output, suggesting that the benefits of probiotics are achieved via reduced illness burden rather than acute physiological enhancement.

Supplement protocols typically range from 10⁸ to 10¹⁰ CFU/day for up to 16 weeks, with multi-strain products combining species like *Lactobacillus rhamnosus*, *Bifidobacterium longum*, and *Streptococcus thermophilus*. While generally well tolerated, probiotics should be introduced only after dietary strategies to improve gut microbiota diversity, such as increased intake of prebiotic-rich foods, have been explored. Prophylactic use 1 to 2 weeks prior to heavy training blocks, travel or competition may help reduce the incidence of URS [126].

Probiotics are not universally effective and may be contraindicated in athletes with persistent GI symptoms at rest or during exercise. These individuals should undergo specialist assessment rather than supplementing with probiotics. Additionally, product quality, viability of strains, and formulation stability vary widely and probiotic efficacy is highly strain specific. Probiotics may support training continuity by enhancing immune resilience and intestinal health. Their use should be individualised, guided by symptom history with support from sports dietitians or healthcare providers.

### 6.10. Protein (Group A) for recovery

Protein supplementation does not directly enhance acute cycling performance, but does play a vital role in supporting recovery, repair and adaptation, especially during periods of heavy training or after extended endurance events [127]. The primary mechanism by which protein benefits endurance athletes is through the stimulation of muscle protein synthesis (MPS), supporting the repair of muscle damage and promoting mitochondrial biogenesis and structural remodelling of muscle fibres [128]. Whey protein, in particular, is rich in essential amino acids especially leucine, a key activator of the mTOR pathway involved in MPS.

Endurance cyclists are advised to consume 20 to 40 grams of high-quality protein following exercise. While the post-exercise carbohydrate window, typically 30 to 120 minutes, is well established for optimising glycogen resynthesis and recovery, the timing of protein intake appears less critical, with no clearly defined “anabolic window” for maximal benefit [129]. Post exercise consumption of protein is especially important following high volume or high intensity training periods. Plant-based alternatives such as soy, rice and pea protein are also effective when consumed in sufficient quantities and may be combined to ensure a complete amino acid profile.

Evidence supports the role of protein supplements in reducing muscle soreness, accelerating recovery and promoting long-term adaptation [130]. By enabling athletes to recover more efficiently, protein supplementation allows for greater training consistency and intensity over time, indirectly enhancing cycling performance through improved resilience and adaptation capacity.

### 6.11. Vitamin C (Group B) for recovery and resilience

Vitamin C (ascorbic acid) is a water-soluble antioxidant and essential micronutrient with emerging or context-specific applications in cycling performance, particularly in mitigating exercise-induced oxidative stress and supporting immune health during periods of intensified training or environmental stress such as altitude training or cold exposure [131].

During prolonged or high-intensity exercise, increased mitochondrial respiration and muscle contraction generate ROS, which may contribute to cellular oxidative damage and fatigue if not adequately neutralised. Vitamin C acts as a direct free radical scavenger and helps regenerate other antioxidants such as Vitamin E, thereby enhancing overall antioxidant capacity [132]. In the context of endurance cycling, where cumulative oxidative stress may impair recovery or promote inflammation, Vitamin C has been proposed as a recovery-supportive supplement.

Supplementation strategies typically involve doses ranging from 200 to 1,000 mg/day, often in combination with other antioxidants. Evidence indicates that Vitamin C supplementation may reduce markers of muscle damage and inflammation including CRP and CK following strenuous exercise [133]. Despite these benefits, caution is warranted regarding chronic high-dose antioxidant use. Several studies have reported that high antioxidant intake, including Vitamin C, may blunt cellular adaptations to endurance training by interfering with redox-sensitive signalling pathways involved in mitochondrial biogenesis including PGC-1α activation [134,135]. Thus, while short-term supplementation may be useful during overload training blocks or competition phases, long-term daily use may not be advisable for all athletes.

### 6.12. Vitamin D (Group A) for health and resilience

Vitamin D plays a critical role in supporting skeletal integrity, muscle function and immune regulation, all of which are physiological domains essential for maintaining training consistency and reducing injury risk in endurance athletes [136]. Although not directly ergogenic, sufficient vitamin D status is crucial for sustaining health and performance capacity over the long term. Deficiency is particularly common among athletes who train predominantly indoors, at high latitudes, or during winter months, with serum 25-hydroxyvitamin D concentrations often falling below the recommended threshold of 30 ng/mL (75 nmol/L).

Vitamin D influences calcium absorption and bone mineralisation, helping to prevent stress fractures, while also modulating neuromuscular function through its action on vitamin D receptors in muscle tissue [137]. Furthermore, it supports immune competence, reducing susceptibility to upper respiratory tract infections during periods of intense training [138]. Supplementation with 1,000 to 2,000 IU/day of vitamin D₃ is generally effective in maintaining adequate circulating levels, though higher doses may be required in cases of deficiency, and individualised dosing based on serum testing is recommended. While vitamin D supplementation does not acutely enhance cycling performance, there is strong evidence that it plays a key supportive role by preserving physiological resilience, reducing training disruptions due to illness or injury, and thereby indirectly contributing to sustained performance adaptations.

### 6.13. Zinc (Group B) for immune function

Zinc is a trace mineral essential for immune function, metabolic processes and gene expression. Approximately 85% of total body zinc is stored in skeletal muscle and bone, highlighting its structural and functional importance. Zinc is not produced endogenously, it must be obtained through the diet, with major sources including meat, seafood, dairy and fortified cereals. Zinc has gained attention as a supplement in cycling primarily for its potential to modulate immune responses, especially during periods of high training load that can suppress immunity [139]. It plays a role in inhibiting the replication of rhinovirus, the most common cause of the common cold, in vitro. Several meta-analyses suggest that zinc supplementation, when initiated early in the course of a cold, may reduce symptom duration by up to 42% [140].

For athletes, supplementation is typically considered for short-term immune support during common colds. Protocols recommend zinc acetate at 75 to 100 mg/day of elemental zinc for five days, initiated within 24 hours of symptom onset. However, this intervention is specific to viral upper respiratory tract infections and not indicated for more serious illnesses like influenza or COVID-19 where misdiagnosis may delay appropriate treatment. Zinc supplementation should not replace medical evaluation for infections with overlapping symptoms. Usage should be supervised, especially in cases of prolonged or recurrent illness.

Lozenges are the most studied form, but absorption varies with different zinc salts and binding compounds. High-dose zinc can cause nausea, constipation, and a metallic taste. Long-term use may lead to copper deficiency due to competitive absorption and formulations with vitamin B6 may increase the risk of peripheral neuropathy, especially if used in conjunction with other vitamin B6 containing supplements [141]. Zinc supplementation may be beneficial in reducing the duration of cold symptoms when used acutely and appropriately. However, evidence for performance enhancement in healthy athletes is limited, and high-dose or prolonged use carries potential risks. Zinc may be used as part of a broader illness management strategy under professional guidance, particularly when symptoms are ambiguous or prolonged.

## 7. Physiological testing in prioritising supplement regimen

To optimise supplementation strategies for cycling performance, it is useful to base interventions on objective physiological assessments that identify individual limitations, deficiencies, or metabolic characteristics (Table 4). Such testing enables the development of targeted, evidence-based protocols that align with specific performance goals, training demands, and health considerations of the individual athlete.

**Table 4.** Physiological testing to prioritise supplement use for cycling performance.

Test	Relevant supplement(s)	Example application(s)	Key use case
VO2max	Carbohydrates, carnitine, dietary nitrates, iron (if deficient), caffeine, exogenous ketones, multivitamin, vitamin D	Low VO2max may indicate the need for carbohydrate loading to increase exercise tolerance. Nitrate supplementation can improve oxygen efficiency; iron can correct anaemia. Carnitine may support fat oxidation during aerobic training. Caffeine and exogenous ketones can boost endurance performance. Multivitamin or vitamin D deficiency correction may support baseline energy metabolism.	Aerobic capacity and endurance profiling
Lactate threshold and blood lactate profile	Beta-alanine, carnitine, creatine, sodium bicarbonate, curcumin, protein, omega-3 fatty acids, *N*-acetylcysteine, vitamin C, calcium	Early lactate rise may benefit from buffering agents like beta-alanine or sodium bicarbonate. Poor recovery suggests creatine or protein intake. Carnitine may reduce lactate. Curcumin, NAC, vitamin C, and omega-3s can modulate inflammation and oxidative stress post-effort. Calcium supports muscle contraction at high intensities.	Buffering strategy for high-intensity efforts
Muscle carbohydrate and fat utilisation testing	Carbohydrates, caffeine, carnitine, exogenous ketones, cherry juice, protein, multivitamin	High carbohydrate reliance at low intensities may benefit from strategic carb intake and caffeine. Carnitine and ketones may improve fat use. Cherry juice can support recovery post-fatigue. Multivitamins ensure micronutrient support for metabolic flexibility.	Fuelling and metabolic efficiency
Sweat rate/electrolyte loss	Electrolytes, glycerol, pickle juice, menthol, zinc	High sweat sodium loss supports personalised electrolyte strategies. Glycerol can aid fluid retention. Pickle juice may reduce cramping. Menthol improves thermal comfort perception. Zinc supports sweat gland function and skin recovery.	Thermoregulation and hydration strategy
Iron panel	Iron (if deficient), vitamin C, multivitamin, probiotics	Low ferritin or haemoglobin guides iron supplementation, ideally co-administered with vitamin C to enhance absorption. Multivitamin ensures co-factors. Probiotics may improve iron absorption via gut health.	Oxygen transport and fatigue prevention
25-hydroxy-vitamin D concentration	Vitamin D, calcium, multivitamin, omega-3 fatty acids	Low vitamin D suggests supplementation to support immunity, bone density, and muscle function. Calcium co-supplementation may improve musculoskeletal integrity. Multivitamins ensure sufficiency of other bone-relevant nutrients. Omega-3s may enhance vitamin D-mediated muscle recovery.	Bone health, immunity, muscle recovery
Omega-3 index	Omega-3 fatty acids, curcumin, vitamin D, multivitamin	Low omega-3 index suggests the need for EPA/DHA supplementation to reduce inflammation, enhance cardiovascular function, and aid muscle repair. Curcumin adds anti-inflammatory support. Vitamin D and multivitamins complement recovery.	Inflammation and recovery support
Recovery biomarkers (e.g. CK, IL-6, cortisol)	*N*-acetylcysteine, omega-3s, protein, vitamin D, curcumin, cherry juice, zinc, collagen, probiotics, calcium	Elevated muscle damage or inflammation markers support increased protein, omega-3s, and vitamin D. NAC and curcumin reduce oxidative stress. Cherry juice, zinc, and collagen aid tissue repair. Calcium supports neuromuscular recovery. Probiotics may improve systemic recovery via gut axis.	Training load management and adaptation
Muscle soreness (subjective or functional testing)	Cherry juice, collagen, protein, curcumin, omega-3s, N-acetylcysteine	Delayed onset muscle soreness (DOMS) may benefit from antioxidant and anti-inflammatory support. Protein aids recovery. Collagen helps tendon/muscle repair.	Post-exercise recovery optimisation
Gastrointestinal symptom screening	Probiotics, multivitamin, electrolytes, curcumin	GI distress during exercise can be improved with targeted probiotics. Multivitamins and curcumin may assist in gut barrier function. Electrolytes reduce osmotic stress.	Gut health and exercise tolerance
Cramping history or neuromuscular irritability	Pickle juice, magnesium (if part of multivitamin), electrolytes, quinine	History of cramping can guide use of pickle juice for rapid relief, electrolytes to reduce imbalance, and quinine (with caution) for nerve excitability modulation.	Cramp management and prevention
Bone density or stress injury history	Calcium, vitamin D, collagen, multivitamin	Athletes with poor bone health may benefit from calcium, vitamin D, and collagen to support bone and connective tissue remodelling.	Bone stress injury risk reduction

One of the foundational assessments is VO₂max testing, which evaluates an athlete’s aerobic capacity. Cyclists with high VO₂max may benefit from carbohydrate supplementation to support sustained aerobic output, while dietary nitrates may improve efficiency by reducing the oxygen cost of submaximal exercise. Caffeine and exogenous ketones may also support endurance by enhancing fat utilisation and cognitive focus during prolonged bouts of exercise.

In athletes presenting with comparatively low or declining VO₂max and symptoms of fatigue, iron status should be assessed via serum ferritin and haemoglobin to evaluate the potential for iron deficiency anaemia, which can impair oxygen transport and limit aerobic performance. In cases of general micronutrient insufficiency, a multivitamin may be used to correct dietary gaps that could subtly impair energy metabolism or red blood cell synthesis. Furthermore, vitamin D may enhance mitochondrial function and immune competence, potentially benefiting endurance performance in vitamin D–insufficient athletes.

Lactate threshold and blood lactate profiling provide valuable insight into anaerobic metabolism and the body's capacity to buffer exercise-induced acidosis [142]. These tests quantify the power intensity at which blood lactate starts to accumulate disproportionately to effort, reflecting a shift from aerobic to anaerobic energy production. Athletes with a low lactate threshold may benefit from beta-alanine supplementation, which enhances intracellular buffering through increased muscle carnosine concentrations, thereby delaying the onset of fatigue during high-intensity efforts [16]. Sodium bicarbonate may further support athletes with rapid lactate accumulation by enhancing extracellular buffering capacity and facilitating the efflux of hydrogen ions from the muscle [85].

Lactate testing can also be used longitudinally to track adaptations to high intensity training and the effectiveness of buffering-related supplementation over time [143]. Carnitine may play a complementary role by enhancing fatty acid oxidation and reducing lactate accumulation during prolonged submaximal efforts.

In athletes demonstrating an inability to sustain repeated high-intensity efforts, creatine monohydrate may support phosphagen system recovery and rapid ATP resynthesis [43]. The use of curcumin, *N*-acetylcysteine (NAC), omega-3 fatty acids, and vitamin C may offer anti-inflammatory and antioxidant support to buffer secondary muscle damage and oxidative stress, while calcium is essential for neuromuscular function and muscle contraction under load.

Metabolic testing, such as indirect calorimetry or lactate profiling, can assess substrate utilisation patterns and inform personalised fuelling strategies [144]. Athletes with a high reliance on carbohydrate oxidation at moderate workloads may prioritise carbohydrate and caffeine supplementation during training and competition. For those seeking to enhance fat oxidation, carnitine, exogenous ketones, and caffeine can be used to promote fatty acid mobilisation and reduce glycogen dependence. Cherry juice may also support recovery in such athletes due to its polyphenol content and antioxidative properties, particularly in low-glycogen training scenarios. Multivitamins ensure adequate B-vitamins and co-factors necessary for mitochondrial metabolism.

In parallel, sweat rate and sodium concentration testing allow for precise electrolyte and fluid replacement strategies, essential for maintaining thermoregulation, hydration, and neuromuscular function during prolonged efforts or in hot environments. Glycerol may be used to enhance fluid retention before events involving heat stress, while menthol can support thermal perception and comfort. Athletes with high sweat sodium concentrations may benefit from pickle juice to help mitigate neuromuscular cramping.

Additionally, zinc plays a supportive role in skin integrity, immune function, and sweat gland activity, making it relevant in high-volume or high-sweat athletes. Routine biochemical testing is also critical for identifying micronutrient deficiencies that can impair performance or recovery. In addition to iron, vitamin C may be used to enhance iron absorption in deficient athletes.

Probiotics can play a supportive role by enhancing gut health and nutrient absorption, including iron and fat-soluble vitamins. Measurement of serum 25-hydroxyvitamin D is advised for cyclists, particularly those with limited sun exposure. A level below 30 ng/mL may necessitate vitamin D supplementation to support bone health, immune function, and muscle recovery. For athletes with a history of bone stress injuries or low bone mineral density, additional calcium and collagen may support connective tissue integrity and skeletal resilience.

Monitoring recovery markers such as heart rate variability (HRV), perceived muscle soreness, and biomarkers of muscle damage (e.g. creatine kinase) can inform the optimal use of protein, omega-3 fatty acids, and vitamin D to promote muscle repair and adaptation. *N*-acetylcysteine, curcumin, and cherry juice can reduce inflammation and oxidative stress during periods of high training load. Collagen and calcium support musculoskeletal recovery, while zinc contributes to tissue repair and immune modulation.

Additionally, athletes experiencing recurrent muscle cramps or neuromuscular irritability may benefit from quinine, electrolytes or pickle juice, used cautiously and based on individual history. Together, these physiological assessments offer a robust and individualised framework for prioritising supplementation strategies. By aligning supplement interventions with test-derived physiological profiles, athletes and practitioners can maximise performance outcomes, enhance recovery, and reduce the risk of nutrient-related performance bottlenecks. This personalised approach fosters not only peak performance but also long-term resilience and health in competitive cycling.

## 8. Discussion

This review has outlined the biochemical foundations of skeletal muscle energy metabolism and provided a detailed rationale for the targeted use of both ergogenic and medical supplements in cycling. The quality and consistency of evidence for individual ergogenic and medical supplements with respect to enhancing cycling performance in the current review support the classifications defined by the AIS framework. The integration of supplement strategies with a nuanced understanding of energy system physiology forms the cornerstone of evidence-based nutritional interventions in both competitive and recreational cycling. Optimising supplementation requires aligning individual metabolic demands with targeted compounds that support specific pathways involved in energy production, buffering, recovery, and resilience.

Skeletal muscle contraction is powered by ATP, regenerated through a network of metabolic pathways including the phosphagen system, glycolysis, oxidative phosphorylation, and *β*-oxidation. These systems are differentially activated based on exercise intensity and duration, and their activity is regulated by substrate availability, enzyme kinetics, mitochondrial function, and acid–base balance. The modulation of these regulatory nodes through supplement use is increasingly recognised as a key strategy to augment performance. For example, creatine monohydrate directly enhances phosphagen system capacity by increasing intramuscular phosphocreatine stores, supporting repeated sprint capacity. Carbohydrates fuel both anaerobic glycolysis and aerobic oxidative phosphorylation, sustaining performance across a broad range of cycling demands. Caffeine and carnitine influence substrate utilisation and central drive, while beta-alanine and sodium bicarbonate modulate pH balance and buffering capacity. These supplements address distinct but interconnected aspects of muscle energetics and fatigue resistance.

The pH sensitivity of key enzymes such as phosphofructokinase-1 (PFK-1) in glycolysis provides mechanistic insight into the efficacy of buffering agents. During high-intensity cycling, the decline in intracellular pH inhibits PFK-1 activity, reducing glycolytic flux. Beta-alanine supports intracellular buffering by increasing muscle carnosine, while sodium bicarbonate enhances extracellular buffering. Their combined use targets both compartments and exemplifies a synergistic approach grounded in biochemical regulation. Oxidative phosphorylation, the backbone of endurance performance, can be modulated by dietary nitrates, which enhance mitochondrial efficiency via nitric oxide-mediated improvements in muscle perfusion. Iron, through its role in haemoglobin and mitochondrial enzymes, is essential for oxygen transport and electron transport chain function, especially in iron-deficient athletes. Carnitine, exogenous ketones, and carbohydrates differentially influence substrate delivery to the mitochondria, enabling flexibility in ATP generation depending on training status and event demands. In ultra-endurance or glycogen-depleted states, *β*-oxidation becomes a dominant energy source. L-carnitine facilitates the transport of long-chain fatty acids into the mitochondria, while exogenous ketones offer an alternative substrate with high oxidative efficiency. These supplements may improve performance in fat-adapted athletes or during multi-hour events where glycogen depletion is a concern. However, the interactions between ketones, carbohydrates, and fat metabolism remain complex and highly context-dependent, underscoring the importance of targeted, rather than blanket, application.

Beyond acute performance enhancement, nutritional strategies must also support recovery, physiological resilience, and long-term adaptation. Protein supplementation is fundamental in promoting muscle repair and mitochondrial biogenesis post-exercise. Omega-3 fatty acids and curcumin help mitigate inflammation and preserve muscle function, especially during intensified training blocks. Vitamin D, calcium, and collagen support bone integrity and musculoskeletal health, crucial in cyclists with low energy availability or those susceptible to stress injuries. Iron, when indicated by biochemical testing, restores oxygen-carrying capacity and mitigates symptoms of fatigue in athletes with iron-deficiency anaemia. Similarly, vitamin C enhances iron absorption, while multivitamins and probiotics can correct or prevent nutrient gaps and improve gut health, respectively—factors often overlooked in endurance athletes with high physiological demand and dietary restrictions. Emerging evidence also suggests that exogenous ketones may support post-exercise recovery by improving glycogen resynthesis and modulating hormonal responses associated with central fatigue. While these findings are preliminary, they highlight the evolving role of supplements that bridge the divide between acute performance and recovery enhancement.

The integration of physiological testing, including VO₂max, lactate threshold, substrate utilisation, sweat composition, and biomarker profiling allows for the development of individualised supplement strategies. These assessments provide actionable insights into aerobic capacity, buffering needs, metabolic efficiency, hydration status, and micronutrient sufficiency. They serve not only as diagnostic tools but also as platforms to evaluate the efficacy of supplement interventions over time. For example, a declining VO₂max with associated anaemia points to iron supplementation, while early lactate accumulation suggests utility for beta-alanine, sodium bicarbonate, or creatine. High sweat sodium losses necessitate personalised electrolyte strategies, potentially including glycerol for pre-loading or pickle juice to mitigate cramps. In the absence of significant musculoskeletal injury, recovery markers such as creatine kinase or HRV can guide the use of protein, omega-3s, vitamin D, and *N*-acetylcysteine to promote adaptation, minimise the recovery period between races and reduce training-induced stress. These test-informed strategies enable practitioners to tailor supplement regimens to an athlete’s unique physiological profile, avoiding unnecessary use and optimising timing, dosage, and context of application. The synergy between diagnostics and supplementation exemplifies the principles of precision nutrition in sports science.

While this review focuses on athletic performance, the physiological principles underpinning supplement efficacy extend to broader clinical and functional settings. The metabolic dysregulation observed in conditions such as cancer-related fatigue—where reliance on glycolysis persists despite oxygen availability (i.e. the Warburg effect)—may benefit from similar metabolic strategies. Supplements that restore mitochondrial function, reduce oxidative stress, or support substrate utilisation (e.g. creatine, ketones, carnitine, NAC) may offer therapeutic potential beyond sport. As such, ongoing research into substrate flexibility, redox modulation, and the interplay between nutrition and gene expression will not only refine athletic supplementation but also contribute to therapeutic strategies in metabolic and neuromuscular disease.

This review synthesises a broad range of literature across both ergogenic and medical supplements, spanning various supplement types, performance contexts and physiological mechanisms. However, several limitations should be acknowledged. First, while efforts were made to prioritise peer-reviewed, human-based research published in English, no formal risk of bias assessment tool (e.g. Cochrane RoB 2.0) was applied due to the heterogeneity of study designs and outcomes. Instead, the strength of evidence for each supplement was assessed qualitatively based on consistency of findings across studies, biological plausibility and alignment with existing consensus classifications (e.g. AIS ABCD system). As such, the quality of included studies may vary, with some exhibiting small sample sizes, lack of blinding or limited ecological validity. Second, many supplements, particularly those in Group B, are supported by promising but incomplete evidence, and findings may not generalise across sex, age, training status or specific cycling disciplines. Third, although we focused on studies published in reputable journals, potential conflicts of interest, particularly in studies evaluating commercially available supplement formulations, were not systematically assessed, and such funding sources may introduce bias in outcome reporting or interpretation. Fourth, the review did not conduct a meta-analysis, and as such, quantitative comparisons between supplements are not possible. Lastly, while we have highlighted individualised approaches based on physiological testing, this review does not account for genetic, hormonal, or microbiome influences that may modulate supplement efficacy. Future research should address these gaps through rigorous, sport-specific trials and stratified approaches that better reflect real-world athletic diversity.

Despite the breadth of evidence synthesised, several important limitations warrant explicit acknowledgement. First, cycling encompasses heterogeneous disciplines spanning track sprinting, criteriums, time trials, road racing and ultra-endurance, each with distinct physiological demands. Consequently, findings from one context may not generalise across all cycling formats. Second, much of the supplementation literature remains male-dominated, limiting confidence in extrapolating efficacy, dosing, and side-effect profiles to female athletes, particularly across different hormonal phases. Third, long-term safety data for several ergogenic supplement, especially those requiring chronic or high dose use such as ketones, NAC and buffering agents, are limited. Most studies focusing on short-term performance outcomes rather than cumulative health effects. Finally, although this review emphasises individualised, test-informed supplementation strategies, robust validation of personalised approaches remains scarce, with few studies directly comparing precision-guided supplementation against standardised protocols. These limitations highlight the need for more diverse, long-duration and context-specific trials to strengthen translation into real-world cycling practice.

The effective use of supplements in cycling is grounded in a deep understanding of energy system biochemistry, physiological regulation, and individualised needs. Group A supplements are supported by strong evidence for efficacy and safety and should form the foundation of ergogenic strategies. Group B supplements offer targeted benefits in specific contexts but require further study. Medical supplements play a critical role in maintaining physiological health and training consistency. Future research should focus on clarifying the context-specific utility of emerging supplements (e.g. ketones, probiotics, NAC), exploring the interactions between supplements and training adaptation, and integrating genomic, microbiomic and metabolomic data to personalise supplementation. This will require collaborative efforts between exercise physiologists, dietitians, clinicians, and molecular biologists to fully realise the potential of nutritional interventions in endurance sport and beyond. Ultimately, supplement use should be framed not as a one-size-fits-all solution but as part of a comprehensive, evidence-informed performance and health strategy that evolves alongside advances in physiology, nutrition, and biotechnology.

## 9. Practical applications and key messages

The evidence presented in this review highlights several important considerations for athletes, coaches and practitioners:

- **Supplements with strong evidence in cycling:** Beta-alanine, caffeine, carbohydrates, creatine monohydrate, dietary nitrates, electrolytes, glycerol and sodium bicarbonate. These supplements are supported by high-quality evidence and can provide meaningful performance benefits when used appropriately.
- **Individualisation is essential:** Supplement strategies should be tailored to the athlete’s characteristics and context, including sex, age, training status, and environmental conditions (e.g. heat, altitude). Physiological testing (e.g. VO₂max, lactate threshold, sweat sodium concentration, micronutrient status) can help guide the selection and timing of supplements.
- **Health, safety and anti-doping considerations:** Only supplements tested in cycling populations and screened by independent third-party programs should be used to minimise health risks and inadvertent doping violations. Athletes must remain vigilant under the WADA strict liability principle, which places full responsibility for substances in their system on the competitor.

In practice, effective supplementation should be viewed as an adjunct and not a replacement for training, nutrition and recovery. Evidence-based, individualised, and ethically sound strategies maximise both performance outcomes and athlete wellbeing.

## Supplementary Material

Supplementary material Supplement_Review_Supplemental_Material.
