# Supplementary material for: A comprehensive review of the physiology and evidence base to guide the use of ergogenic and medical supplements for enhanced cycling performance
Source: J Int Soc Sports Nutr. 2026 Feb 13;23(1):2630487. doi: 10.1080/15502783.2026.2630487 (PMC12912213; doi:10.1080/15502783.2026.2630487)
Supplement: Supplementary material — Supplement_Review_Supplemental_Material. [file RSSN_A_2630487_SM3141.docx]

## Supplemental Table 1:Synthesis and interpretation of key recent reviews and meta-analyses defining the evidence for the use of ergogenic supplements in cycling

| **Supplement** | **AIS Group** | **Citation** | **Studies in Analysis** | **Event** | **Population** | **Performance Type** | **Main Finding** | **GRADE Certainty** | **NHMRC Grade** | **Interpretation** |
| --- | --- | --- | --- | --- | --- | --- | --- | --- | --- | --- |
| **Beta-alanine** | **A** | Saunders, 2017 (1) | 40 | Various inc. cycling | Trained & recreational | High intensity performance | Improved performance in 1 to 4 min efforts | Moderate | B | Consistent benefit in high-intensity exercise capacity (1–4 min efforts), especially in cycling time trials and track events. Evidence weaker for longer aerobic–anaerobic transition efforts, but overall moderate-certainty support. |
|  |  | Huerta Ojeda, 2020 (2) | 19 | Various inc. cycling | Trained & recreational | Aerobic–anaerobic transition | Improved time trial performance and time to exhaustion |  |  |  |
| **Caffeine** | **A** | Chen, 2024 (3) | 15 | Cycling | Trained | Time trial | Improved time trial performance and power output | Moderate-High | A | Robust and consistent benefit for endurance performance, particularly cycling time trials. Strongest evidence at 3–6 mg/kg doses; low doses (<3 mg/kg) less effective. High applicability to both cycling and broader endurance sport. |
|  |  | Southward, 2018 (4) | 46 | Various inc. cycling | Trained & recreational | Endurance performance | Improved endurance performance for doses of 3-6 mg/kg but not <3mg/kg |  |  |  |
| **Carbohydrate** | **A** | Vandenbogaerde, 2011 (5) | 73 | Various inc. cycling | Trained & recreational | Endurance performance | Improved time trial performance and time to exhaustion | Moderate | B/C | Clear performance benefits (3–6%) in endurance events when optimal protocols are used (multiple boluses, glucose polymers ± fructose/protein). Strongest evidence for prolonged cycling time trials; effects less reliable with suboptimal feeding strategies. |
|  |  | Pöchmüller, 2016 (6) | 24 | Predominantly Cycling  (21/24 studies) | Trained & recreational | Time trial | Improved time trial performance and power output |  |  |  |
| **CHO Mouth Rinse** |  | Brietzke, 2019 (7) | 13 | Cycling | Trained | Time trial | Increased power output but no effect on time trial performance | Moderate | B |  |
| **Carnitine** | **B** | Ayuso, 2021 (8) | 11 | Various inc. cycling | Trained & recreational | Performance at multiple intensities | Improved performance for high (>80% VO_2_max) but not moderate intensity | Low | C | Strong biochemical evidence (↑ serum carnitine, VO₂max) but inconsistent translation to performance. Possible small benefits in high-intensity (>80% VO₂max) or sprint-type efforts, but low-certainty overall. |
|  |  | Vecchio, 2021 (9) | 30 | Various non-cycling | Trained & recreational | Energy metabolism | Increased VO_2_max and serum carnitine, but no change in lactate |  |  |  |
| **Creatine Monohydrate** | **A** | Forbes, 2023 (10) | 13 | Various inc. cycling | Trained & recreational | High intensity performance | Improved performance in high intensity surges diminishing with duration | High | A | High-certainty evidence of ergogenic benefit for short-duration, high-intensity efforts (<3 min), including cycling sprints and repeated surges. Robust improvements in lower-limb strength across populations. |
|  |  | Lanhers, 2015 (11) | 60 | Various inc. cycling | Trained & recreational | Lower limb strength | Improved lower limb strength performance for effort <3 min |  |  |  |
| **Dietary Nitrate** | **A** | Tan, 2024 (12) | 7 | Cycling | Trained & recreational | High intensity performance | Improved time-to-peak power but no effects on power output metrics | Low | C/D | Evidence shows improvements in exercise capacity (time-to-exhaustion) and sprint acceleration (time-to-peak power), but little to no effect on time-trial performance. Benefits may be context-specific (sub-elite, high-intensity, or hypoxic settings). Certainty very low. |
|  |  | McMahon, 2017 (13) | 76 | Various inc. cycling | Trained & recreational | Endurance performance | Improved endurance capacity limited effect on time trial |  |  |  |
| **Electrolytes** | **A** | Holland, 2017 (14) | 9 | Cycling | Trained & recreational | Endurance performance | Improved performance in moderate intensity efforts longer than 60 minutes | Moderate | B/C | Fluids and electrolytes improve endurance performance in efforts >60 min, particularly in heat, by maintaining hydration status and plasma sodium. May impair 1 h high-intensity efforts. Certainty moderate–low. |
|  |  | Rowlands, 2022 (15) | 28 | Various inc. cycling | Trained & recreational | Central hydration | Electrolytes ingested during exercise provide the greatest benefit to hydration. |  |  |  |
| **Exogenous Ketone** | **B** | Valenzuela, 2020 (16) | 13 | Various inc. cycling | Trained & recreational | Exercise performance | Did not significantly improve overall exercise or time trial performance | Very Low | C/D | Very limited and inconsistent evidence; no reliable improvement in time-trial or endurance performance. Effects equivocal in both cycling and other modalities. Very low certainty. |
|  |  | Margolis, 2020 (17) | 10 | Predominantly Cycling  (8/10 studies) | Trained & recreational | Lower body power and endurance performance | Equivocal effects on both lower body power and endurance performance |  |  |  |
| **Glycerol** | **A** | Goulet, 2007 (18) | 14 | Various inc. cycling | Trained & recreational | Endurance performance | Improved endurance performance and hydration status | Moderate | B | Strong evidence for improved fluid retention; performance benefits inconsistent. May provide endurance benefits in hot/prolonged cycling events where hydration is critical. Overall certainty moderate for hydration, very low for performance. |
|  |  | Van Rosendal, 2009 (19) | 22 | Various including cycling | Trained & recreational | Exercise performance | Improved time trial performance and time to exhaustion |  |  |  |
| **Menthol** | **B** | Jeffries, 2019 (20) | 11 | Various inc. cycling | Trained & recreational | Time trial and thermal sensation | Improved exercise performance and thermal sensation especially with internal application | Very Low | C/D | Internal application (mouth rinse/ingestion) consistently improves thermal sensation and may provide small performance gains in hot environments. External application (sprays/creams) shows little effect. Certainty very low. |
|  |  | Gavel, 2024 (21) | 10 | Predominantly Cycling  (8/10 studies) | Trained & recreational | Exercise performance | Did not significantly improve overall exercise capacity and performance |  |  |  |
| **N-Acetylcysteine** | **B** | Rhodes, 2017 (22) | 7 | Various inc. cycling | Trained & recreational | Exercise performance | Did not significantly improve overall exercise capacity and performance | Very Low | C/D | Evidence does not support consistent performance enhancement. Robust antioxidant effects (↓ oxidative stress, ↑ glutathione) may aid recovery or performance in long-duration, multistage cycling. Very low certainty for direct ergogenic effects. |
|  |  | Fernández-Lázaro, 2023 (23) | 16 | Various inc. cycling | Trained & recreational | Exercise performance and biomarkers | Improved exercise performance, antioxidant capacity and glutathione homeostasis |  |  |  |
| **Sodium Bicarbonate** | **A** | Lopez-Silva, 2023 (24) | 18 | Cycling | Trained | Time trial | Improved time trial performance and power output | Moderate | B | Moderate-certainty evidence that individualized ingestion protocols improve short-duration high-intensity performance (~+3% power, −1% TT time), particularly in cycling time trials and track efforts. Strong biochemical effects on buffering capacity. |
|  |  | Calvo, 2022 (25) | 17 | Various inc. cycling | Trained & recreational | Energy metabolism | Improved pH, HCO3, base excess and blood lactate |  |  |  |

**Reference**

1. Saunders B, Elliott-Sale K, Artioli GG, Swinton PA, Dolan E, Roschel H, et al. β-alanine supplementation to improve exercise capacity and performance: a systematic review and meta-analysis. Br J Sports Med. 2017;51(8):658-69.

2. Huerta Ojeda Á, Tapia Cerda C, Poblete Salvatierra MF, Barahona-Fuentes G, Jorquera Aguilera C. Effects of Beta-Alanine Supplementation on Physical Performance in Aerobic–Anaerobic Transition Zones: A Systematic Review and Meta-Analysis. Nutrients. 2020;12(9):2490.

3. Chen B, Ding L, Qin Q, Lei T-H, Girard O, Cao Y. Effect of caffeine ingestion on time trial performance in cyclists: a systematic review and meta-analysis. Journal of the International Society of Sports Nutrition. 2024;21(1):2363789.

4. Southward K, Rutherfurd-Markwick KJ, Ali A. The Effect of Acute Caffeine Ingestion on Endurance Performance: A Systematic Review and Meta–Analysis. Sports Medicine. 2018;48(8):1913-28.

5. Vandenbogaerde TJ, Hopkins WG. Effects of Acute Carbohydrate Supplementation on Endurance Performance. Sports Medicine. 2011;41(9):773-92.

6. Pöchmüller M, Schwingshackl L, Colombani PC, Hoffmann G. A systematic review and meta-analysis of carbohydrate benefits associated with randomized controlled competition-based performance trials. Journal of the International Society of Sports Nutrition. 2016;13(1):27.

7. Brietzke C, Franco-Alvarenga PE, Coelho-Júnior HJ, Silveira R, Asano RY, Pires FO. Effects of Carbohydrate Mouth Rinse on Cycling Time Trial Performance: A Systematic Review and Meta-Analysis. Sports Medicine. 2019;49(1):57-66.

8. Mielgo-Ayuso J, Pietrantonio L, Viribay A, Calleja-González J, González-Bernal J, Fernández-Lázaro D. Effect of Acute and Chronic Oral l-Carnitine Supplementation on Exercise Performance Based on the Exercise Intensity: A Systematic Review. Nutrients. 2021;13(12):4359.

9. Vecchio M, Chiaramonte R, Testa G, Pavone V. Clinical Effects of L-Carnitine Supplementation on Physical Performance in Healthy Subjects, the Key to Success in Rehabilitation: A Systematic Review and Meta-Analysis from the Rehabilitation Point of View. Journal of Functional Morphology and Kinesiology. 2021;6(4):93.

10. Forbes SC, Candow DG, Neto JHF, Kennedy MD, Forbes JL, Machado M, et al. Creatine supplementation and endurance performance: surges and sprints to win the race. Journal of the International Society of Sports Nutrition. 2023;20(1):2204071.

11. Lanhers C, Pereira B, Naughton G, Trousselard M, Lesage F-X, Dutheil F. Creatine Supplementation and Lower Limb Strength Performance: A Systematic Review and Meta-Analyses. Sports Medicine. 2015;45(9):1285-94.

12. Tan R, Cass JK, Lincoln IG, Wideen LE, Nicholl MJ, Molnar TJ, et al. Effects of Dietary Nitrate Supplementation on High-Intensity Cycling Sprint Performance in Recreationally Active Adults: A Systematic Review and Meta-Analysis. Nutrients. 2024;16(16):2764.

13. McMahon NF, Leveritt MD, Pavey TG. The Effect of Dietary Nitrate Supplementation on Endurance Exercise Performance in Healthy Adults: A Systematic Review and Meta-Analysis. Sports Medicine. 2017;47(4):735-56.

14. Holland JJ, Skinner TL, Irwin CG, Leveritt MD, Goulet EDB. The Influence of Drinking Fluid on Endurance Cycling Performance: A Meta-Analysis. Sports Medicine. 2017;47(11):2269-84.

15. Rowlands DS, Kopetschny BH, Badenhorst CE. The Hydrating Effects of Hypertonic, Isotonic and Hypotonic Sports Drinks and Waters on Central Hydration During Continuous Exercise: A Systematic Meta-Analysis and Perspective. Sports Medicine. 2022;52(2):349-75.

16. Valenzuela PL, Morales JS, Castillo-García A, Lucia A. Acute Ketone Supplementation and Exercise Performance: A Systematic Review and Meta-Analysis of Randomized Controlled Trials. International Journal of Sports Physiology and Performance. 2020;15(3):298-308.

17. Margolis LM, O'Fallon KS. Utility of Ketone Supplementation to Enhance Physical Performance: A Systematic Review. Advances in Nutrition. 2020;11(2):412-9.

18. Goulet EDB, Aubertin-Leheudre M, Plante GE, Dionne IJ. A Meta-Analysis of the Effects of Glycerol-Induced Hyperhydration on Fluid Retention and Endurance Performance. International Journal of Sport Nutrition and Exercise Metabolism. 2007;17(4):391-410.

19. Van Rosendal SP, Osborne MA, Fassett RG, Coombes JS. Physiological and performance effects of glycerol hyperhydration and rehydration. Nutrition Reviews. 2009;67(12):690-705.

20. Jeffries O, Waldron M. The effects of menthol on exercise performance and thermal sensation: A meta-analysis. J Sci Med Sport. 2019;22(6):707-15.

21. Gavel EH, Barreto G, Hawke KV, Stellingwerff T, James LJ, Saunders B, et al. How Cool is That? The Effects of Menthol Mouth Rinsing on Exercise Capacity and Performance: A Systematic Review and Meta-analysis. Sports Medicine - Open. 2024;10(1):18.

22. Rhodes K, Braakhuis A. Performance and Side Effects of Supplementation with N-Acetylcysteine: A Systematic Review and Meta-Analysis. Sports Medicine. 2017;47(8):1619-36.

23. Fernández-Lázaro D, Domínguez-Ortega C, Busto N, Santamaría-Peláez M, Roche E, Gutiérez-Abejón E, et al. Influence of N-Acetylcysteine Supplementation on Physical Performance and Laboratory Biomarkers in Adult Males: A Systematic Review of Controlled Trials. Nutrients. 2023;15(11):2463.

24. Lopes-Silva JP, Correia-Oliveira CR. Acute effects of sodium bicarbonate ingestion on cycling time-trial performance: A systematic review and meta-analysis of randomized controlled trials. European Journal of Sport Science. 2023;23(6):943-54.

25. Calvo JL, Xu H, Mon-López D, Pareja-Galeano H, Jiménez SL. Effect of sodium bicarbonate contribution on energy metabolism during exercise: a systematic review and meta-analysis. Journal of the International Society of Sports Nutrition. 2021;18(1):11.
